# Genome assembly and population genomic analysis reveal the genetic basis of popcorn evolution

**DOI:** 10.1111/pbi.70125

**Published:** 2025-05-05

**Authors:** Xiaojian Fang, Hangqin Liu, Jiacheng Liu, Yang Song, Min Xu, Xing Jian, Li Dong, Qianwen Zhang, Le Xu, Guorui Fan, Zhaoying Wang, Yiwen You, Tianyu Feng, Wenyu Li, Yuling Li, Rentao Song, Zhongwei Lin

**Affiliations:** ^1^ State Key Laboratory of Maize Bio‐Breeding, National Maize Improvement Center, Department of Crop Genetics and Breeding China Agricultural University Beijing China; ^2^ Sanya Institute of China Agricultural University Sanya Hainan China; ^3^ State Key Laboratory of Wheat and Maize Crop Science, Collaborative Innovation Center of Henan Grain Crops, College of Agronomy Henan Agricultural University Zhengzhou China

**Keywords:** popcorn, genome sequencing, kernel poping, domestication

## Abstract

Popcorn, one of the world's most popular snack foods, represents the most ancient type of maize domesticated by humans. However, the genetic basis underlying popcorn evolution and kernel‐popping traits remains largely unknown. In this study, we assembled a high‐quality genome sequence of the popcorn landrace *Strawberry Popcorn* (SP) and conducted extensive population genomic analyses. The SP genome spans 2.3 Gb and harbours a large inversion on chromosome 8, along with millions of genetic variants that enable the discovery of beneficial alleles. Translocations and substantial duplications of the *Ga1* gene occurred in the locus associated with unilateral cross‐incompatibility on chromosome 4. Tandemly duplicated *Ga1* genes underwent pseudogenisation and truncation with complete loss of gene function. The *P1* gene experienced gene expansion and regulatory modifications, leading to downregulation of transcription and subsequent loss of pericarp colour during maize domestication and improvement. Population genomic analysis further identified a subset of 12 marker genes from over 2494 genes under human selection, which were reshaped to enhance kernel‐popping traits during domestication. These marker genes include *Pl1* and *Dek1* for pericarp and aleurone layer thickness; *THP9*, *Sh2*, *SUS1*, *Smk10*, *KW1*, *O7*, and *NKD1* for protein and starch biosynthesis; and *VP5*, *CCD7*, and *Crti3* for carotene biosynthesis, which all influence endosperm vitreousness, a key factor determining kernel hardness for popping. Among these genes, *KW1* and *O7* stand out as pivotal genes with a significant impact on kernel‐popping performance. These results provide a wealth of gene targets to greatly accelerate the molecular breeding of improved popcorn varieties.

## Introduction

Maize (*Zea mays*) is one of the most important crops globally, with an annual production exceeding one billion tonnes (https://www.fao.org), and it has been a genetic model system for over a century. Maize was domesticated from its progenitor teosinte in Mexico approximately 10 000 years ago (Doebley *et al*., 2006). After thousands of years of domestication, diversification, and improvement, maize has accumulated a vast amount of phenotypic diversity. Maize is classified into several types based on kernel characteristics, which are largely influenced by the composition and arrangement of starch in the endosperm. The main types of maize are dent corn, flint corn, sweet corn, popcorn, flour corn, and waxy corn (Reddy *et al*., 2012). Popcorn is often considered the oldest form of maize, as early maize specimens discovered at numerous prehistoric archaeological sites have predominantly been identified as belonging to this variety (Grobman *et al*., 2012). Among the different maize types, popcorn is a unique variety of maize that has been extensively studied for its distinctive popping ability and genetic characteristics. Popcorn has a unique combination of a hard outer shell and dense starchy interior, allowing it to pop when heated. It is specifically cultivated for popping quality. Apart from kernel‐popping traits, popcorn generally exhibits tillering, small kernels, and relatively more kernel rows.

With the development of high‐quality genome sequencing technologies, numerous maize genome sequencing projects have been completed. The first maize genome to be sequenced was B73, an important parent in maize breeding and the primary reference genome for maize. This B73 genome has now been updated to version 5 (Hufford *et al*., 2021). Another significant parent, Mo17, has been sequenced to produce a high‐quality T2T (telomere‐to‐telomere) assembly, making it the first complete telomere‐to‐telomere maize genome (Chen *et al*., 2023). An important NAM (Nested Association Mapping) population in maize, consisting of 26 parents, has also been subjected to high‐quality sequencing and pan‐genome analysis, representing the largest pan‐genome sequencing project in maize to date (Hufford *et al*., 2021). Additionally, four representative European varieties (F7, EP1, DK105, and PE0075) have been sequenced to high quality, along with 12 major inbred lines from China (Haberer *et al*., 2020; Wang *et al*., 2023). The genomes of sweet corn and a local variety, SK, have also been sequenced (Hu *et al*., 2021; Yang *et al*., 2019). Despite the substantial number of completed maize genome sequencing projects, there is still a scarcity of sequenced genomes for landraces. Landraces contain superior alleles that are valuable for breeding purposes, making it essential to sequence and analyse their genomes.

**Figure 1:**
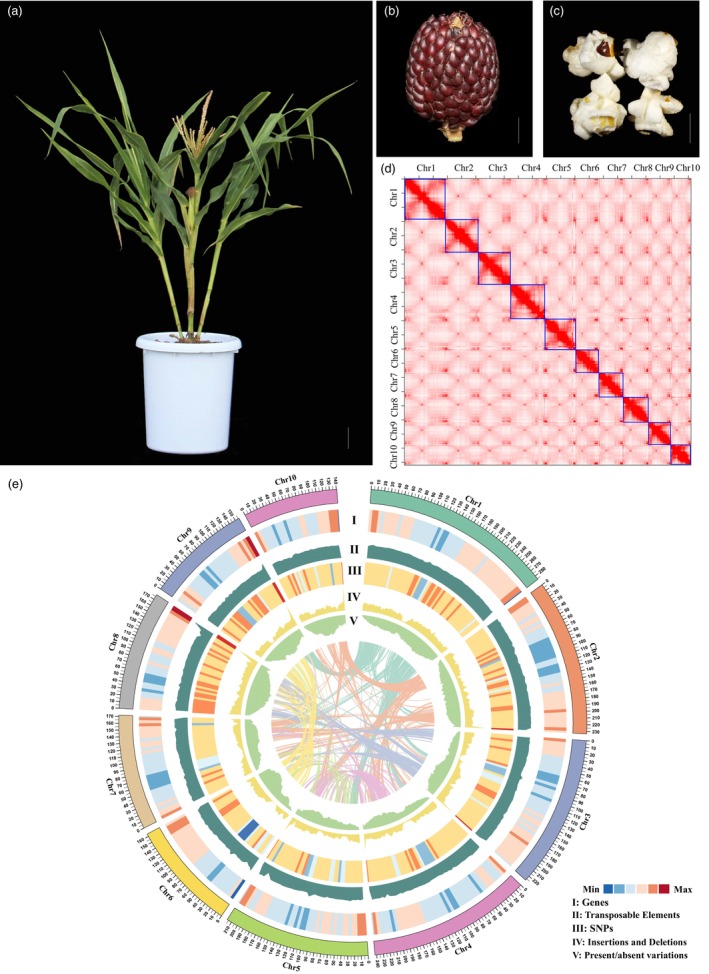
Phenotypes of SP and an overview of the genome assembly of SP. (a–c) The phenotypes of a popcorn landrace strawberry popcorn (SP): SP exhibits tillering (a), a strawberry‐like ear (b), and kernels that pop when heated (c). (d) Hi‐C contact heatmap for the assembled chromosomes. (e) Circle views of the SP genome. The circles, from outer to inner, represent the densities of genes (I), transposable elements (II), SNPs (III), insertions and deletions (IV), and presence/absence variants (PAVs) (V).

In this study, we assembled a high‐quality genome sequence of the landrace popcorn variety SP from America. This landrace is characterised by its dwarf stature, tillering, small kernels with a brown‐red colour, and more than 20 kernel rows (Figure 1a–c and Figure S1). SP contains a 2.3‐Gb genome sequence, and comparative genomic analysis revealed a large inversion on chromosome 8, as well as millions of genetic variants. The locus of unilateral cross‐incompatibility on chromosome 4 experienced translocation and frequent duplications of the *Ga1* gene, leading to pseudogenisation and loss of function. The *Pericarp color1* (*P1*) gene was also duplicated and underwent regulatory changes, resulting in the downregulation of its transcription during maize domestication and improvement. A large‐scale population genomic analysis identified 2494 genes under human selection in popcorn, among which a subset of core genes for kernel‐popping traits was discovered. *KW1* and *O7*, as the representative genes, significantly impact kernel‐popping capability. These core genes will be invaluable for developing new popcorn through molecular breeding in the future.

## Results

### High‐quality assembly of the genome of the popcorn landrace SP

To assemble the SP genome, we sequenced SP through PacBio Sequel II and Illumina Hiseq Hi‐C (Hi‐C) platforms, which generated a total of 58 Gb (~26.93‐fold coverage) of HiFi long reads (Wenger *et al*., 2019) and 192 Gb Hi‐C reads (~144.43‐fold coverage) (Table S1). These HiFi reads were self‐corrected and assembled using hifiasm, resulting in 2513 contigs with an N50 of 59.68 Mb (Cheng *et al*., 2021) (Table S1). To further anchor the super scaffolds and unscaffolded contigs into pseudochromosomes, Hi‐C mapping was employed for scaffolding through a layered clustering strategy (Putnam *et al*., 2016). The Hi‐C assembly was subsequently polished using short‐read Illumina data. The final assembly has a genome length of 2.3 Gb and includes 10 long super scaffolds, referred to as pseudochromosomes (Figure 1d), with a total length of 2.04 Gb, as well as 3976 unassigned contigs with a total length of 283 Mb (Table S2). The pseudochromosomes covered 87.82% of the total assembly with a scaffold N50 of 217.1 Mb (Table S2) (Chor *et al*., 2009).

The quality and completeness of the popcorn genome were evaluated using BUSCO and Long Terminal Repeat (LTR) Assembly Index (LAI) analyses (Ou *et al*., 2018; Simão *et al*., 2015). The BUSCO analysis revealed that 96.4% (1555) of the Plantae BUSCO genes were present in the assembled popcorn genome as complete, 2.2% as fragmented, and 1.4% as missing (Table S3). Among the complete genes, 86.60% were single‐copy and 9.80% were duplicated (Table S3). These results are comparable to those obtained for field corn reference genomes, such as the B73 v5 genome (Hufford *et al*., 2021). The LAI score, which indicates the proportion of intact LTR sequences in the genome, was used to assess the assembly's continuity and completeness. The popcorn genome assembly achieved a mean LAI score of 24.27, classified as ‘gold’ quality, signifying high continuity and completeness of our assembly (Ou *et al*., 2018).

### Genome annotation

Repetitive sequence annotation revealed that a total of 1.93 Gb of sequences were identified as repetitive, constituting 83.44% of the genome (Figure 1e and Tables S2 and S3). Among these, LTR retrotransposons accounted for the largest proportion, representing 65.43% of the entire genome (Table S4). Within this category, Gipsy‐type elements were the most abundant at 43.49%, followed by Copia‐type elements at 12.29% (Table S4). DNA transposons constituted 18% of the repetitive sequences, with helitron‐type elements being the most prevalent at 12.11% (Table S4). The content of repetitive sequences in the SP genome is comparable to those in other reported maize genomes.

Protein‐coding gene annotation was performed using a combination of de novo annotation, transcript‐assisted annotation, and homology‐based annotation methods. Full‐length transcripts were obtained from samples collected from the root, stem, leaf, tassel, and silk tissues during the pollen‐shedding stage. This comprehensive approach led to the identification of 42 572 genes, with 37 493 of these genes annotated on chromosomes (Figure 1e and Tables S2 and S5). The average gene length was 2804 base pairs (bp), and 38 993 genes were functionally annotated using the InterProScan database (https://www.ebi.ac.uk/interpro/about/interproscan/). We compared the gene number among SP, MS71, and B73 (Figure S3). Most of the genes (38011) were shared among these three genomes. Only 2775 genes were specific to SP, and the number of specific genes in B73 and MS71 was 286 and 2150, respectively. This result suggests that the annotated 42 572 protein‐coding genes are reliable.

### Genome comparisons

**Figure 2:**
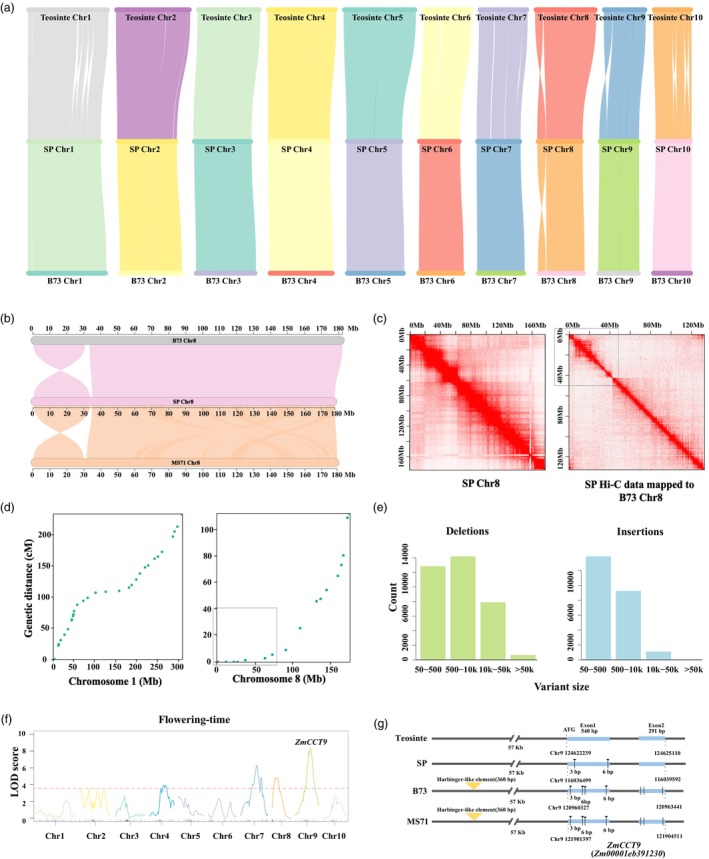
Genome comparisons. (a) Genome collinearity among the maize wild progenitor teosinte, SP, and B73. (b) Genome comparison revealed a large inversion on chromosome 8. (c) The large inversion on chromosome 8 is shown based on Hi‐C data mapped onto the SP and B73 genomes. The inversion is highlighted in a box. (d) Genetic distance of a maize chromosome, using chromosome 1 as an example. The genetic distance of the long arm of chromosome 8 (0–50 Mb) is nearly zero due to the large inversion. (e) The distribution of variants in the SP genome. (f) QTL mapping of flowering time in the SP‐MS71 F_2_ population. The horizontal black line represents the threshold of the significant logarithm of the odds (LOD) at the *P* = 0.05 level. (g) Causal variant analysis of the major QTL of flowering time on chromosome 9, corresponding to *CCT9*. The transposon is absent in SP and teosinte, while present in MS71 and B73. The orange triangle indicates the insertion of the Harbinger‐like element.

Large inversions are frequently present between the SP and teosinte genomes; however, inversions are greatly reduced between the SP and domesticated maize MS71 genomes (Figure 2a). Interestingly, we observed a 30‐Mb inversion on chromosome 8 compared to the B73 and MS71 genomes (Figure 2b). To confirm the accuracy of this inversion, we aligned Hi‐C data to the B73 reference genome (Figure 2c). This analysis corroborated the presence of this inversion (Figure 2c). The large inversion on chromosome 8 is consistent with the genetic distance of this region in the F_2_ population (Figure 2d), which was constructed from the cross between SP and MS71 (Figure S1). Recombination generally occurs more frequently at both ends than in the middle of each chromosome (Figure 2d). However, nearly no recombination was present at the end of the long arm of chromosome 8 (0–50 Mb), and the genetic distance at positions from 0 to 50 Mb is nearly zero (Figure 2d). This result suggested that no recombination occurs in this large fragment because of the inversion at the end of the long arm of chromosome 8. Furthermore, to investigate the occurrence of this inverted region in other genomes, we compared it with the 25 genomes from nested association mapping populations (NAM) parents and found that most of these genomes also exhibited this inversion (Figure S2) (Buckler *et al*., 2009). This large inversion does indeed affect recombination; no recombination was observed in this large inversion in the F_2_ population. RNA‐seq data showed that genes in this large inversion exhibited a normal pattern compared to other regions. Furthermore, no significant structural variants were present, and single‐nucleotide polymorphism (SNP) density was normal in this inversion, and only one quantitative trait locus (QTL) for leaf width was detected in this region. These facts signify that this large inversion might not impair gene expression.

Genome comparisons identified a significant number of SNPs and insertions/deletions (InDels) in the SP genome. Specifically, when compared to B73 (v5), we detected a total of 27 526 763 SNPs and 2 333 497 InDels (Figures 1e and 2e). Among these SNPs, 753 721 (1.87%) were located in exon regions, 2 696 697 (6.68%) in intron regions, and 5 431 790 (13.45%) in upstream regions. A total of 22% of all these SNP and InDel variants were located in gene regions. Additionally, we identified 59 969 presence‐absence variants (PAVs) ranging from 50 bp to 1 Mb between SP and B73 (Table S6). As expected, the majority of these PAVs were relatively short, with 44.60% being less than 500 bp and only 0.39% exceeding 100 kb. The average length of these PAVs was 6.73 kb, with approximately 8000 located in gene bodies and promoter regions. We next performed QTL mapping for flowering time in the SP‐MS71 F_2_ population and identified three major QTLs of flowering time on chromosomes 7, 8, and 9 (Figure 2f and Figure S1e,f). The QTL on chromosome 9 co‐located with the previously reported *CCT9* (Huang *et al*., 2018). Sequence comparison between SP and MS71 showed that MS71 contained a 360‐bp transposable insertion while SP lacked this insertion at the causal site of *CCT9* (Figure 2g). The QTL of flowering time on chromosome 10, which corresponds to *CCT10*, represents the QTL of flowering time with the strongest genetic effect in maize NAM populations (Buckler *et al*., 2009). However, this QTL on chromosome 10 was not detected in this study (Figure 2f). Further sequence analysis revealed that both SP and MS71 harboured the causal transposon variant in the promoter of *CCT10* (Figure S4) (Yang *et al*., 2013). Therefore, the strongest QTL of flowering time is absent in the SP‐MS71 F_2_ population. These results suggest that the abundant variants identified in the SP genome can greatly facilitate the cloning of QTLs for complex traits.

### Evolution of the locus of unilateral cross‐incompatibility on chromosome 4 in maize

**Figure 3:**
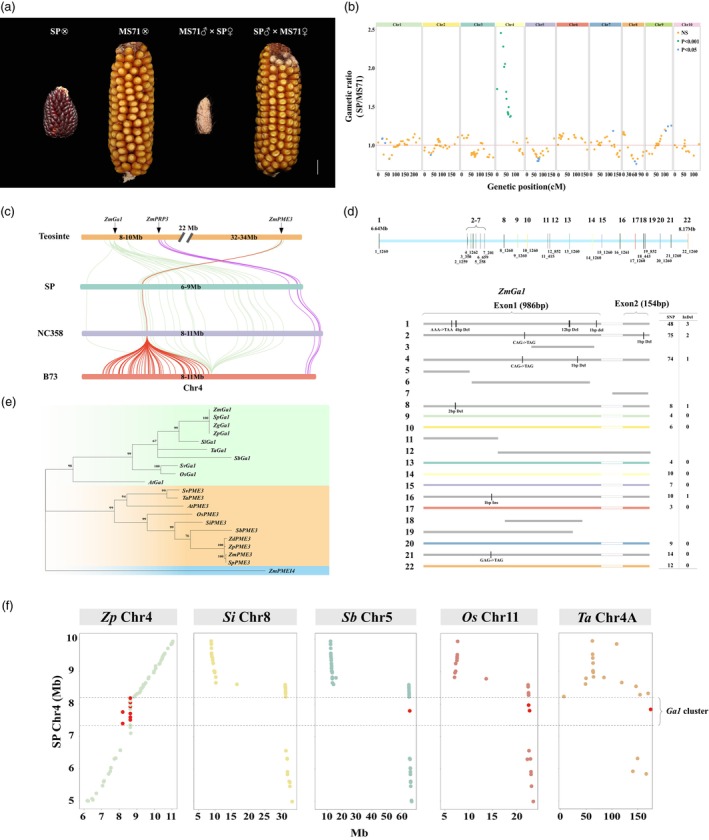
The evolution of the unilateral cross‐incompatibility locus in maize. (a) Unilateral cross‐incompatibility of SP. SP is sterile when crossed with MS71 but remains fertile when self‐pollinated. In contrast, MS71 is fertile when crossed with SP or when selfing. (b) A strong segregation distortion occurs on chromosome 4. Green, blue, and orange dots represent the significance of the segregation ratio deviating from the expected normal gametic ratio of 1 (SP/MS71): *P* < 0.001, 0.05, and non‐significant (NS), respectively. The red line signifies the expected normal gametic ratio of 1 (SP/MS71). (c) Evolution of the copy number of the three key genes *ZmGa1*, *ZmPME3*, and *ZmPRP3* in the unilateral cross‐incompatibility locus. SP, NC358, and B73 represent the *Ga1‐S*, *Ga1‐M*, and *Ga1* haplotypes, respectively. Green, red, and purple lines indicate the changes in *ZmGa1*, *ZmPME3*, and *ZmPRP3* copy numbers among teosinte, SP, NC358, and B73. (d) Structure and copy number of the *ZmGa1* gene in SP. The number of *ZmGa1* copies is shown above chromosome 4 (thick blue line), and the length of each gene copy is displayed below chromosome 4. The gene structures of *ZmGa1* copies are shown using filled (exon) and blank (intron) boxes in different colours, with black lines on the boxes signifying the variants that induce the loss of function of *ZmGa1*. (e) Phylogenetic tree for *ZmGa1* and *ZmPME3*, both of which encode pectin methylesterase, using *ZmPMEI4* as the outgroup. (f) Genome collinearity in the unilateral cross‐incompatibility locus among teosinte (Zp), SP, foxtail millet (Si), sorghum (Sb), rice (Os), and wheat (Ta). The *Ga1* cluster is highlighted in the dashed‐line box, and red dots represent the orthologous genes of *ZmGa1*.

Maize unilateral cross‐incompatibility (UCI) is a pre‐zygotic reproductive barrier known to restrict gene flow between maize and teosinte and among maize populations (Jones, 1924; Kermicle, 2006). SP exhibits cross‐incompatibility when it is crossed with MS71. When SP is used as the pollen donor, it can fertilise MS71 successfully, resulting in normal seed set. However, when popcorn serves as the female parent, it cannot accept pollen from MS71, thus failing to produce seeds (Figure 3a). To investigate which genomic region is responsible for the unilateral cross‐incompatibility in SP, we genotyped 286 individual plants from the F_2_ population between SP and MS71. Most maize genomic regions showed normal segregation of SP and MS71 alleles (Figure 3b). However, the region from 1 Mb to 30 Mb on chromosome 4 displayed a strongly significant segregation distortion in this F_2_ population, where the genotypes of the region from the 286 F_2_s significantly deviate towards SP (Figure 3b). This region coincides with the *Gametophyte factor1* (*Ga1*) locus, which has been associated with UCI in previously reported studies (Jiménez and Nelson, 1965; Zhang *et al*., 2018). In previous studies, MS71 was identified as the *ga1* haplotype, while popcorn was classified as the *Ga1‐S* haplotype (Jones and Goodman, 2018). The *Ga1* locus contains three tightly linked genes: the male determinant *ZmGa1* (*Zm00001eb167600*), which is expressed in pollen; the female determinant *ZmGa1F/ZmPME3*; and the promoter of pollen tube growth *ZmPRP3* as the braker of *ZmPME3*, both are expressed in silks (Moran Lauter *et al*., 2017; Wang *et al*., 2022; Zhang *et al*., 2023). There are three distinct haplotypes associated with the *Ga1* locus: *ga1* haplotypes, *Ga1‐S* haplotypes, and *Ga1‐M* haplotypes (Jones and Goodman, 2018). The *Ga1‐S* haplotype can pollinate both *Ga1‐M* haplotypes and *ga1* haplotypes, but *ga1* haplotypes cannot pollinate *Ga1‐S* haplotypes. In contrast, the *Ga1‐M* haplotype can both accept and donate pollen to all haplotype types (Jones and Goodman, 2018).

To investigate how these three determinants of unilateral cross‐incompatibility function in maize evolution, we compared the *Ga1* genomic regions among teosinte (Ames21814), SP (*Ga1‐S* haplotype) and other maize including NC358 (*Ga1‐M* haplotype) and B73 (*ga1* haplotype) (Figure 3c) (Huang *et al*., 2022; Hufford *et al*., 2021). The *Ga1* locus is situated in the regions of 8 Mb and 31–33 Mb, and the *PME3* is located in the region of 33 Mb on chromosome 4 in teosinte (Figure 3c). In contrast, the two regions harbouring multiple *Ga1* copies and a single copy of *PME3* are merged into the single region spanning from 6 Mb to 9 Mb on chromosome 4 in SP (Figure 3c). The *PME3* in the region from 31 to 33 Mb was translocated to the region of 8 Mb during maize domestication (Figure 3c). The genomic fragment from 8 Mb to 33 Mb on chromosome 4 in teosinte maintained a normal linear order compared with that in SP (Figure 3c).

We next carefully compared the copies of *Ga1*, *PME3*, and *PRP3* genes among these genomes. Teosinte contains 29 copies of *Ga1*, a single copy of *PME3*, and four copies of *PRP3* (Figure 3c). 18 of 29 *Ga1* copies have a complete coding sequence (CDS) with full gene function (Figure S5). The remaining 11 copies of *Ga1* include four copies encoding normal‐length CDSs with variants inducing loss of function and seven truncated copies lacking gene function (Figure S4). Tandem duplicated genes have several evolutionary fates, which include pseudogenisation, conservation of gene function, loss of function, subfunctionalisation, and even neofunctionalisation. These 18 tandem *Ga1* genes with complete CDS maintain the gene function, while the four copies of *Ga1* with loss‐of‐function variants (three 1‐base pair (bp) deletions and two 1‐bp insertions) and seven truncated copies exhibit pseudogenisation. The exons of 22 full *Ga1* copies in teosinte harbour a total of 171 SNPs and five InDels (Figure S6). The SP genome has 22 copies of *ZmGa1* segments, a single copy of *PME3*, and four copies of *PRP3* (Figure 3c). Among the 22 *Ga1* copies, only eight contain complete CDSs with full gene function (Figure 3d). The resting six copies contain variants (three early stoppage mutations and seven InDels) inducing loss of function, and eight copies are truncated (Figure 3d and Figure S6). The exons of 14 full *Ga1* copies in SP contain a total of 191 SNPs and eight InDels (Figure 3d). In comparison to SP (*Ga1‐S* haplotype), the B73 and MS71 genomes (*ga1* haplotype) have lost most copies of *Ga1* (Figure 3c) and retain only one copy of *Ga1* with a longer sequence causing a premature translation stoppage (Zhang *et al*., 2018), along with a rapid expansion of truncated *PME3* short segments (Figure 3c) and harbouring all four copies of *PRP3*. Both *Ga1* and *PME3* in the same locus encode genes in the pectin methylesterase family. Phylogenetic analysis revealed that both *Ga1* and *PME3* belong to parallel clades (Figure 3e).

We also compared the genomic region on chromosome 4 containing the *Ga1* locus across various crops, including rice, wheat, foxtail millet, and sorghum (Figure 3f). The flanking regions surrounding the *Ga1* gene cluster exhibited strong collinearity (Figure 3f). However, the *Ga1* gene's dramatic expansion, with over ten copies, is only present in teosinte and popcorn (Figure 3f). The *Ga1* gene is absent on chromosome 8 in foxtail millet, duplicated once in rice, and remains as a single copy in wheat (Figure 3f). This result indicates that the *Ga1* gene underwent significant duplication in maize during cereal evolution.

### The evolution of the *P1* gene in maize

**Figure 4:**
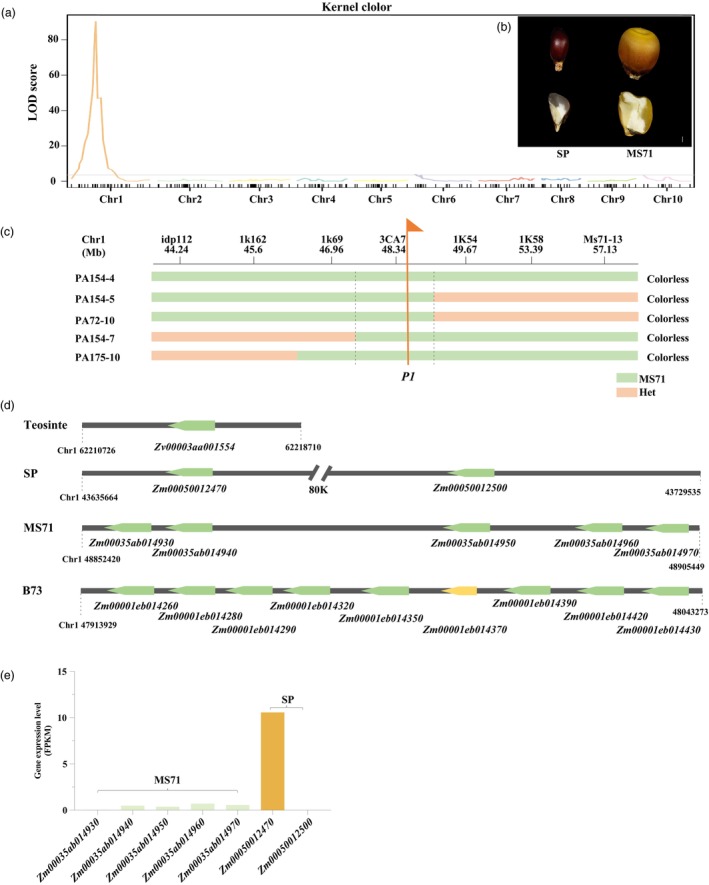
Evolution of the *Pericarp color1* (*P1*) gene. (a) A major QTL for pericarp colour was identified in the F_2_ population derived from crosses between SP and the elite inbred line MS71. (b) SP shows a brown‐red pericarp, while MS71 exhibits a colourless pericarp. (c) Fine mapping revealed that the *Pericarp color1* (*P1*) corresponds to the major QTL for pericarp colour. All these recombinant plants exhibited a colourless pericarp. The flag represents the target gene *P1*. Yellow bars represent genotypes from MS71 with a colourless pericarp, while pink bars signify the heterozygous genotypes. (d) The changes in copy number of *P1* among teosinte, SP, MS71, and B73. Green and orange bars indicate the *P1* gene with coding sequences (CDSs) with full function and partial loss of function, respectively. (e) Transcription levels of the *P1* genes in maize kernels from SP and MS71 as measured by RNA‐seq. The transcription levels were quantified in fragments per kilobase of transcript per million fragments mapped (FPKM).

The SP exhibits brown‐red kernels, while the MS71 variety has yellow kernels (Figure 4). To identify the gene responsible for the pericarp colour change between the parents, we performed QTL mapping in the SP‐MS71 F_2_ population. We identified one major QTL associated with pericarp colour located on chromosome 1 (Figure 4a,b). The QTL on chromosome 1 has a LOD score of 88.22 and explains 74.5% of the phenotypic variation, with a peak near marker 3CA7 (48 Mb based on the B73v4 reference genome) (Figure 4c). This QTL for pericarp colour was then narrowed down to within 3 Mb based on four recombination plants from the F_2_ population. This region contains the cloned gene *P1* (48 589 178 bp) for maize pericarp colour (Grotewold *et al*., 1994). The *P1* (*Zm00001eb014290*) gene encodes an R2R3 MYB transcription factor that regulates the synthesis of red flavonoid pigments in the pericarp and other floral tissues (Zhang and Peterson, 2005).

To further investigate whether the *P1* gene controls pericarp colour between the parents, we compared the genomic sequence in this locus. Genome comparisons revealed that the SP has two copies of the *P1* gene (Figure 4d). In contrast, MS71 harbours five copies of the *P1* gene, in which four copies have a complete coding sequence (CDS) and one copy contains a CDS with a 1‐bp insertion and a 1‐bp deletion, leading to a frame shift (Figure 4d and Figure S7). The wild progenitor teosinte has only one single *P1* gene (Figure 4d). Meanwhile, B73 contains nine copies, all have complete CDS, except for the fifth copy with a 1‐bp insertion and a 1‐bp deletion, causing a frame shift (Figure 4d and Figure S8). By comparing 26 NAM founder inbred lines, we found that tropical maize, popcorn, and sweet corn typically have fewer copies of *P1*, whereas Stiff‐Stalk lines show higher copy numbers. These results further support our hypothesis that *P1* experienced copy number expansion during maize domestication and improvement, with modern maize inbred lines generally having a higher copy number of *P1*.

To identify how the *P1* genes control the pericarp colour in maize, we analysed their expression using RNA sequencing (RNA‐seq) from the samples of SP and MS71 kernels 18 days after pollination, when the pericarp of SP exhibited light purple. The first *P1* copy was strongly expressed, while nearly no expression of the second copy was detected in SP kernels (Figure 4e). In contrast, all five copies of *P1* were expressed at extremely low levels in MS71 kernels (Figure 4e). Although MS71 has five copies of *P1*, this increased copy number did not significantly enhance total transcription levels, suggesting that regulatory changes in the *P1* genes repressed their expression levels in MS71. Therefore, the greatly increased expression of the *P1* gene in SP leads to the synthesis of more red flavonoid pigments, resulting in brown‐red pericarp colour.

### Popcorn shows a closer phylogenetic relationship to the wild progenitor teosinte compared to common maize

**Figure 5:**
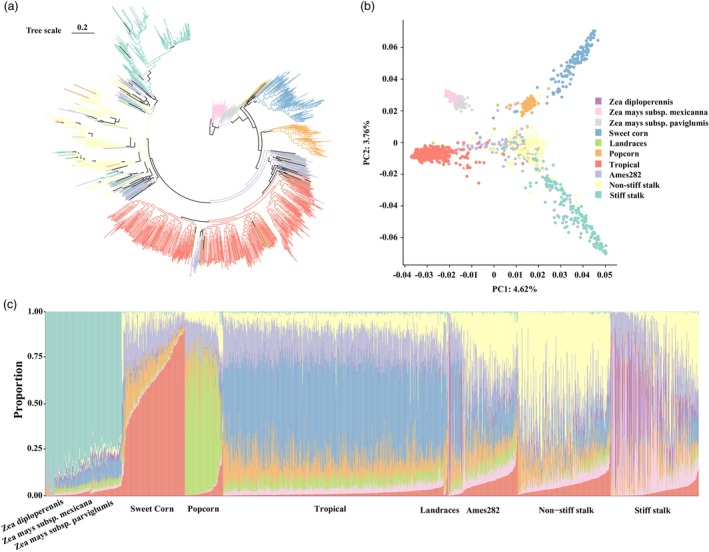
Population genetic structure analysis. (a) Phylogenetic tree of 1468 samples based on SNPs from genotyping by sequencing, using *Zea diploperennis* as the outgroup. (b) Principal component analysis (PCA) of these accessions. (c) Structure analysis of these accessions, conducted using the program STRUCTURE with an assumed number of genetic clusters (*K* = 9), reveals clear evidence of admixture among the accessions.

To explore how the popcorn evolved during maize domestication and improvement, we applied genotyping‐by‐sequencing data from 1468 diverse maize accessions to produce a maximum likelihood‐based phylogenetic tree using RaXML‐NG (Figure 5a and Table S7) (Chen *et al*., 2022; Romay *et al*., 2013; Swarts *et al*., 2017). All maize accessions, including sweet corn, popcorn, landrace, and common maize, descended from the teosinte clade, including *Z. mexicana*, *Z. parviglumis*, and *Z. diploperennis* (Figure 5a). Compared to common maize, sweet corn and popcorn with the SP were located closer to the clade of maize progenitor, teosinte, in this phylogenetic tree (Figure 5a). The phylogenetic relationships were further supported by principal component analysis (PCA) (Figure 5b). Common maize is clearly split into tropical, non‐stiff‐stalk, and stiff‐stalk clusters (Figure 5a,b). Next, structure analysis revealed nine subgroups for these maize accessions, and sweet corn, popcorn, and common maize subgroups showed admixture (Figure 5c).

### The genes under selection in popcorn during maize domestication

**Figure 6:**
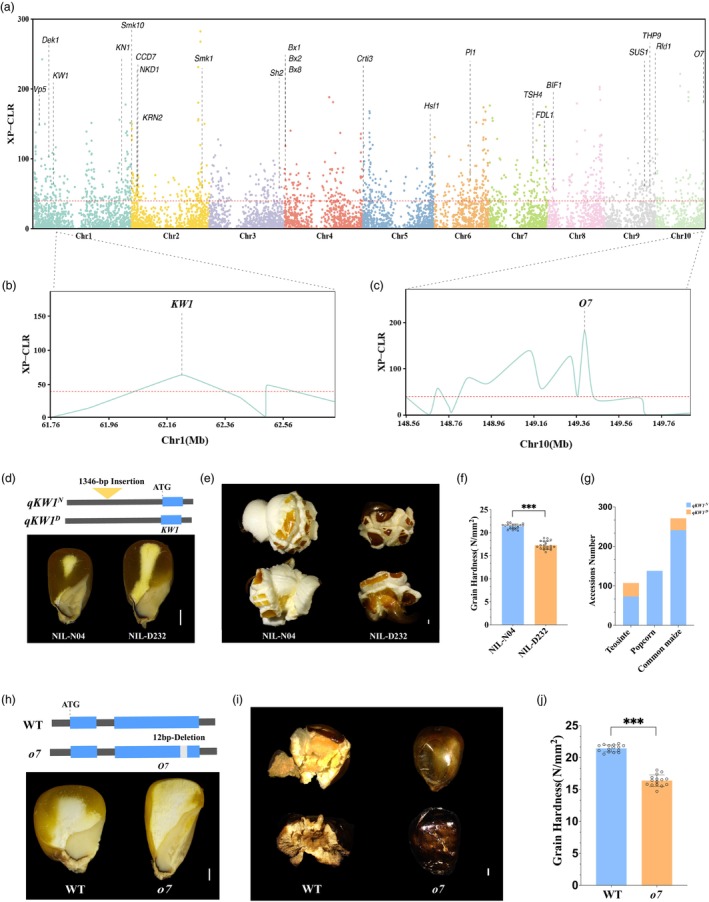
Genome‐wide nucleotide diversity and selection in popcorn. (a) Selective sweeps across the genome were identified between teosinte and popcorn using the XP‐CLR (cross‐population composite likelihood ratio) test. The red dashed line represents the threshold of the top 5% XP‐CLR value, and regions surpassing the XP‐CLR value over the threshold are recognised as selective. The cloned marker genes under selection are highlighted. (b) Detailed views of two selective regions containing *KW1* and O7 are shown. (c) *KW1* is a major QTL for kernel weight between dent maize (D232) and popcorn (N04). N04 harbours a 1346‐basepair (bp) insertion in the promoter of *KW1* (*qKW1* ^*N*^), while this insertion is absent in the promoter of *KW1* in D232 (*qKW1* ^*D*^). (d) The near‐isogenic line N04 (NIL‐N04) contains more vitreous endosperms in kernels than NIL‐D232. (e) Kernels of the NIL‐N04 display a larger expansion volume than those of NIL‐D232 when kernels are heated. (f) The NIL‐N04 exhibits a harder kernel than the NIL‐D232. (g) The distribution of the *qKW1* ^*N*^ allele with the 1346‐bp insertion of *KW1* in teosinte, popcorn, and common maize populations. (h) The 12‐bp deletion in the *O7* gene results in a floury endosperm phenotype. (i) Kernels from the *o7* mutant pop upon heating, unlike wild‐type kernels. (j) The *o7* mutant exhibits softer kernels compared to the wild type. Scale bar, 1 mm. ***P < 0.001.

To investigate the putatively selected regions between teosinte and popcorn in our germplasms, the cross‐population composite likelihood ratio (XP‐CLR) selective sweep parameter was calculated. Genomic regions with the top 5% XP‐CLR values for each comparison were considered the most highly selected genomic regions during the domestication of popcorn. In total, there were 2494 genes located in 1154 highly differentiated genomic regions surpassing the top 5% XP‐CLR threshold between teosintes and popcorn (Figure 6a and Table S8). We then performed KEGG pathway enrichment analysis of these selected genes. This analysis revealed that only five pathways including glycosylphosphatidylinositol (GPI)‐anchor biosynthesis, glycosaminoglycan degradation, N‐glycan biosynthesis, benzoxazinoid biosynthesis, and various types of N‐glycan biosynthesis passed the significance threshold (*P* < 0.05) in the top 25 KEGG enrichment pathways (Figure S10).

Among these selected genes, we found several cloned genes related to growth and development (Figure 6a and Table S9), including *Knotted 1* (*Kn1*), *Tassel sheath 4* (*Tsh4*), *Hairy sheath frayed 1* (*Hsf1*), *Rolled leaf 1* (*Rld1*), *Fused leaves 1* (*Fdl1*), *Kernel row number 2* (*KRN2*), and *Barren inflorescence 1* (*Bif1*). *Kn1*, encoding a transcription factor, plays a key role in the establishment and maintenance of plant meristems, which can greatly impact plant and ear architectures in maize (Bolduc and Hake, 2009). *Tsh4* encodes an SBP‐box transcription factor and controls the development of bracts and the formation of meristem boundaries. It was recently reported to be involved in maize domestication, including the improvement of yield and reshaping of plant architecture (Chuck *et al*., 2010; Xie *et al*., 2024; Zhang *et al*., 2024). *Hsf1* encodes a cytokinin receptor, *Histidine Kinase 1*, and controls leaf patterning and hair formation (Muszynski *et al*., 2020). *Rld1*, which contains an HD‐ZIPIII domain, plays an important role in maize leaf adaxial cell fate (Juarez *et al*., 2004). *Fdl1* encodes a MYB domain protein and activates a conserved regulatory network for cuticular wax biosynthesis in plants (La Rocca *et al*., 2015). Popcorn typically exhibits more kernel rows in comparison to other types of maize. *KRN2*, which encodes a WD domain protein, may contribute significantly to increased kernel rows in popcorn during maize domestication. *Bif1*, as a key component of auxin signal transporter, controls the development from inflorescence meristem to spikelet pair meristem, which directly regulates maize kernel row numbers (Barazesh and McSteen, 2008). Additionally, *Kn1* may contribute to increased kernel rows in popcorn (Bolduc *et al*., 2012; Vollbrecht *et al*., 1991).

A set of genes related to plant metabolism was involved in the domestication process from teosinte to popcorn (Figure 6a and Table S9). These genes include *Benzoxazinless 1* (*Bx1*), *Benzoxazinone synthesis 2* (*Bx2*), *Benzoxazinone synthesis 8* (*Bx8*), *Purple plant 1* (*Pl1*), Viviparous 5 (*Vp5*), *Carotenoid Cleavage Dioxygenase* 7 (*CCD7*) and *Carotene isomerase 3* (*Crti3*). Benzoxazinoids, as basic metabolites, defend maize plants against various microbial pathogens and insect herbivores (Frey *et al*., 2009). *Bx1*, *Bx2*, and *Bx8* function as key enzymes in the biosynthesis of benzoxazinoids in maize. Anthocyanin pigmentation benefits plants by aiding in their adaptation to biotic and abiotic stresses (Frey *et al*., 1997; Tzin *et al*., 2017; Von Rad *et al*., 2001). *Pl1*, encoding a MYB domain, is central to regulating the synthesis of anthocyanin in maize (Gross and Hollick, 2007). The anthocyanin is deposited in the maize pericarp, which will enhance pericarp thickness. *Vp5*, *CCD7*, and *Crti3* are involved in the biosynthesis of carotenoids in maize endosperm (Booker *et al*., 2004; Hable *et al*., 1998; Owens *et al*., 2014). Carotenoids greatly impact the kernel texture; more carotenoids will significantly enhance popcorn kernel hardness (Wang *et al*., 2020).

Popcorn is typically characterised by its popped kernels. The hardness of the endosperm makes a major contribution to popping ability (Vázquez‐Carrillo *et al*., 2019). The hardness of kernels is influenced by pericarp thickness and many physical and chemical properties of the kernel in popcorn. Popcorn's kernels generally consist of a predominantly vitreous endosperm and a smaller proportion of floury endosperm (Figure 4b). Vitreous endosperms, which contain higher levels of protein (zein), amylose, and damaged starch are harder than floury endosperm (Xu *et al*., 2019). More cloned marker genes related to starch and protein synthesis were identified from these 2494 selected genes in popcorn (Figure 6 and Table S9). The *Teosinte high protein 9* (*THP9*) gene is a key regulator of high protein content, encoding the protein *asparagine synthetase 4* (*ASN4*), and variations in the *THP9* gene are also associated with improved nitrogen use efficiency and increased biomass in maize plants (Huang *et al*., 2022). Three genes including *Shrunken 2* (*Sh2*), *Sucrose Synthase 1* (*SUS1*), and *Small kernel 10* (*Smk10*) were selected for the reshaping of starch in popcorn. *Sh2* encodes ADP‐glucose pyrophosphorylase, which represents the rate‐limiting starch biosynthetic enzyme in starch biosynthesis (Bhave *et al*., 1990; Manicacci *et al*., 2007). Overexpression of *SUS1* can greatly enhance grain yield and increase amylose content (Amor *et al*., 1995; Li *et al*., 2023). *Smk10* encodes a choline transporter‐like protein that regulates the development of plasmodesmata (PD) in transfer cells and the starch content in kernels (Hufford *et al*., 2021).

Only one gene, *Defective kernel 1* (*Dek1*) is involved in the development of the pericarp (aleurone layer) in popcorn (Figure 6a and Table S9). The *Dek1* mutant exhibited chalky endosperm lacking an aleurone layer (Becraft *et al*., 2002). Three genes containing *Kernel weight 1* (*KW1*), *Opaque7* (*O7*), and *Naked endosperm 1* (*NKD1*) play dual roles in regulating protein (zein) synthesis and starch biosynthesis (Figure 6a–c and Table S9). *NKD1* can interact with *Naked endosperm 2* (*NKD2*) and the core gene for zein, *Opaque 2* (*O2*), to regulate the switch from cellular development to the biosynthesis and filling of starch and protein in maize endosperm (Wu *et al*., 2024). The *KW1* gene corresponds to a QTL of kernel weight between a popcorn line, N04, and a dent maize line, Dan232 (Zhang *et al*., 2024). *KW1* encodes an E3 ubiquitin protein ligase with a SINA domain. A 1.3‐kb insertion in the promoter of *KW1* upregulates its transcription and enhances endosperm vitreousness (Figure 6d). The gene *KW1* is also located within the major QTL of popping in the population between the popcorn N04 and the dent line Dan232 (Li *et al*., 2009; Zhang *et al*., 2024). The kernel of the near‐isogenic line of N04 (NIL‐N04) is significantly harder than that of NIL‐Dan232 (Figure 6d,f). The protein content of the kernel is enhanced, whereas the starch content is decreased in the NIL‐N04 compared with the NIL‐Dan232. Therefore, the kernel expansion volume of NIL‐N04 is clearly larger than that of NIL‐Dan232 when heated (Figure 6e). Overexpression of *KW1* resulted in a reduced floury endosperm and a harder kernel in comparison to the control plant with normal expression of *KW1*. Kernels of the overexpression plant showed a larger expansion volume than the control plant when heated (Figure S9). However, the kernel popping ability from the *KW1* overexpression plant under dent corn background was much weaker than that of NIL‐N04 under popcorn genetic background.

Further investigation of the 1.3‐kb insertion in the promoter of *KW1* revealed that this insertion was present in 72 out of 106 teosinte accessions, all 138 popcorn accessions (Zhang *et al*., 2024) and 240 out of 270 common maize accessions (Figure 6g and Table S10). Teosinte *KW1* harbours both the insertion and deletion of the 1.3‐kb sequence in the promoter (Table S10), signifying that the causal variant in *KW1* is a standing variation. This standing variation in *KW1* may have been gradually accumulated and eventually become fixed in popcorn under selection during domestication. *O7* encodes an acyl‐activating enzyme and controls the development of protein bodies and starch content and ultimately impacts endosperm vitreousness and kernel expansion volume as well (Miclaus *et al*., 2011). Partial loss of function of *O7* in the mutant results in floury endosperm, and the kernel hardness is significantly lowered in the mutant compared to the wild‐type plant (Figure 6h,j). The lower kernel hardness in the *o7* mutant results in the failure of kernel popping when heated (Figure 6i). These results indicate that *KW1* and *O7* played important roles in the reshaping of kernel popping during domestication.

## Discussion

### High‐quality genome sequence of SP facilitates discovering favourable alleles from landraces and broadens the genetic diversity of maize elite germplasm

Maize was domesticated from teosinte approximately 10 000 years ago and has undergone an extensive evolutionary journey. Initially, maize was domesticated into landraces, which were subsequently improved into elite maize lines through human selection. However, this process imposed severe genetic bottlenecks or selection sweeps, which drastically reduced DNA diversity (Wang *et al*., 2017). Modern elite maize germplasm originates from a limited number of landraces and has been further developed into distinct heterotic groups, retaining an estimated 60%–70% of the nucleotide diversity found in teosinte (Eyre‐Walker *et al*., 1998; Tenaillon *et al*., 2004). Strong selection pressures from advanced cycle breeding for target traits such as stress resistance may lead to the loss of favourable alleles in maize elite germplasm.

This loss of genetic diversity has constrained the improvement of hybrid maize yields recently. In contrast, maize landraces preserve over 80% of the DNA diversity present in teosinte. These landraces, which were adapted to diverse environments, harbour a wealth of favourable alleles that were lost during the development of modern elite lines. One such allele is the *Tunicate 1* (*Tu1*) allele found in the SP popcorn landrace in this study, characterised by a 5‐bp insertion in the distal promoter region (Li *et al*., 2024). This insertion upregulates *Tu1* transcription, leading to a moderate increase in the number of leaves above the ear, which ultimately enhances maize yield. Unfortunately, this advantageous allele was lost during the improvement of elite maize lines.

In this study, we successfully assembled a high‐quality genome sequence of SP (Figure 1). This resource holds immense potential to rediscover lost favourable alleles, such as *Tu1*, and reintroduce them into elite maize germplasm. By leveraging the genetic diversity of landraces like SP, we can address the limitations of modern maize and significantly improve yields by broadening genetic diversity.

### Complex gene regulatory patterns of kernel popping in popcorn

Popcorn exhibits the amazing trait of kernel popping, driven by the interaction between water content and its unique kernel structure. A popcorn kernel typically consists of an outer vitreous endosperm encasing an inner floury endosperm, all surrounded by a hard pericarp. This structure acts as a pressure vessel during heating. When the kernel is exposed to heat, water within the vitreous endosperm and especially the floury endosperm rapidly vaporises into steam, creating pressure inside until the kernel explosively pops.

Popping volume is influenced by several factors, which include kernel water content, size, shape, and hardness (Vázquez‐Carrillo *et al*., 2019). Appropriate water content is crucial, and deviations from the optimal range significantly lower popping efficiency (Hoseney *et al*., 1983). Small, round kernels incline to yield higher popping volumes, whereas kernel hardness plays a central role in determining expansion capacity. Harder kernels produce larger expansion volumes because they can provide higher pressure for explosives in kernels during heating (Hoseney *et al*., 1983). Kernel hardness is generally associated with the thickness of the pericarp and aleurone layer and the endosperm vitreousness (Vázquez‐Carrillo *et al*., 2019). Vitreous endosperm harbours a higher content of proteins such as zein and amylose and has starch with larger granule sizes compared to floury endosperm (Xu *et al*., 2019).

**Figure 7:**
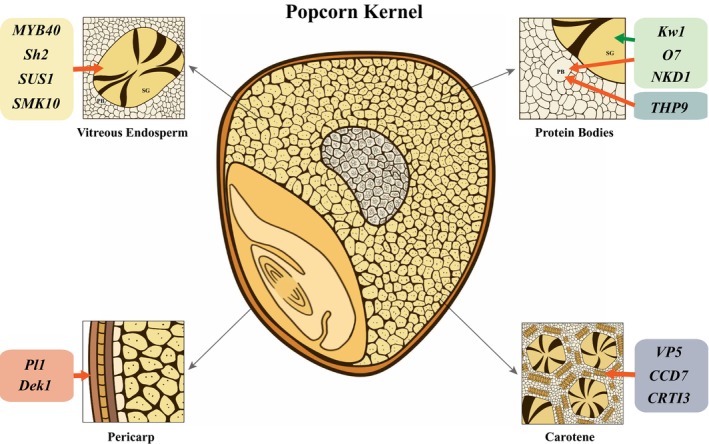
The regulatory gene pattern for kernel hardness in popcorn. Several factors that affect kernel hardness in popcorn include the protein, starch, and carotene contents of the endosperm, as well as the thickness of the pericarp and aleurone layer. *THP9*, *Sh2*, *SUS1*, and *Smk10* control the synthesis of proteins and starch, while *KW1*, *O7*, and *NKD1* coordinate the synthesis of both proteins and starch. *Vp5*, *CCD7*, and *Crti3* are key regulators of carotene synthesis. *Pl1* regulates the synthesis of anthocyanin, which accumulates in the pericarp and aleurone layer. In contrast, *Dek1* governs the development of aleurone layer cells. This complex gene regulatory pattern was reshaped to enhance kernel‐popping capacity in popcorn during domestication. In popcorn cross‐section kernel, floury endosperm (in white) is surrounded by vitreous endosperm (in orange). PB, protein body; SG, starch granule.

In this study, XP‐CLR analysis identified 2494 genes under significant selection during the domestication of popcorn. Among these, a subset of key genes was cloned and found to play pivotal roles in reshaping kernel size, shape, and hardness, which are critical for the popping ability (Figure 6). Genes such as *KRN2*, *Bif1*, and *Kn1*, which are associated with kernel row number and ear development, may influence grain size and shape. For instance, an increase in kernel rows generally results in smaller kernel sizes. *Pl1* regulates anthocyanin synthesis, causing the pigment to accumulate in the pericarp and aleurone layer (Figure 7). More anthocyanin in both the pericarp and aleurone layer will enhance the thickness of the kernel surface. Also, *Dek1* governs the development of aleurone layer cells and may contribute to its thickness. Carotene synthesis, influenced by genes such as *Vp5*, *CCD7*, and *Crti3*, enhances endosperm vitreousness through increased carotene content (Figure 7). Other genes, including *THP9*, *Sh2*, *SUS1*, and *Smk10*, regulate the synthesis of proteins and starch to affect endosperm vitreousness (Figure 7). In contrast, *KW1*, *O7*, and *NKD1* coordinate the synthesis of both proteins and starch to further impact endosperm vitreousness (Figure 7). This complex gene regulatory network was likely shaped by human selection for over millennia in popcorn during maize domestication (Figure 7). Human selection on these marker genes gradually transformed the physical and chemical properties of popcorn kernels, optimising popping characteristics. Despite genetic factors controlling the popping ability, environmental factors such as temperature, drought, and moisture can impair it. It is certain that the interaction between genetic and environmental factors controls popping ability (Sweley *et al*., 2012). These environmental factors should affect popcorn kernel chemical and physical composition and then affect popcorn kernel popping ability. However, how environmental factors control this popping ability remains largely unknown.

This study's identified marker genes for kernel popping will significantly accelerate the development of new popcorn varieties with enhanced popping performance through molecular breeding.

## Methods and Materials

### Plant materials

The F_2_ population with 286 individuals derived from the crosses between SP and MS71 was planted with a 50‐cm row‐to‐row and 25‐cm plant‐to‐plant distance in the experimental station of China Agricultural University in Beijing in 2019. Phenotypic data were collected from this F_2_ population in 2019.

### SP genome sequencing and assembly

High‐quality genomic DNA was extracted from fresh leaves of SP plants for sequencing. SMRTbell libraries were constructed following the standard protocol provided by Pacific Biosciences and sequenced on the PacBio Sequel II platform to produce HiFi reads. Hi‐C libraries were prepared using the established in situ DLO Hi‐C protocol and sequenced by paired ends (2 × 150 bp) on the Illumina HiSeq X‐Ten platform. To assemble the genome of SP, PacBio HiFi reads were first assembled into contigs using hifiasm (v.0.16.1) under ‐l 0 mode. Hi‐C reads were then mapped to the assembled contigs with the juicer pipeline (v.1.5.7), and scaffolded by 3D‐DNA (v1.8) with assembly mode (‐r 0) (Dudchenko *et al*., 2017; Durand *et al*., 2016). Manual curation of duplications and phasing errors was carried out using Juicebox (v1.13.01), and the final chromosome‐level genome was generated using 3D‐DNA. The completeness and quality of the genome assembly were evaluated using BUSCO (v5.2.2), based on lineage‐specific benchmarking datasets (Simão *et al*., 2015).

### Genome annotation

Transposon elements (TEs) in the SP genome were annotated using the EDTA tool (v1.9.5), leveraging the Maize TE Consortium (MTEC; https://github.com/oushujun/MTEC) (Ou *et al*., 2019). To mask the genome, RepeatMasker (v4.0.8; http://www.repeatmasker.org/) was employed, and then the unmasked genome sequence was utilised for protein‐coding gene prediction. Protein‐coding gene models were predicted by integrating three distinct approaches: ab initio prediction, homology‐based search, and transcriptome‐based prediction. Ab initio gene prediction was conducted using Augustus (v2.4) with default parameters (Stanke *et al*., 2006). For homology‐based annotation, GenomeThreader (v1.7.1) was applied, using genomic data from six plant species: *Arabidopsis thaliana*, *Oryza sativa*, *Setaria italica*, *Sorghum bicolor*, *Triticum aestivum*, and *Zea mays*, which were subsequently integrated with CD‐HIT (v4.6) (Fu *et al*., 2012). Expression data based on isoform‐sequencing (Iso‐seq) were collected from five distinct tissues: root, leaf, stem, seed, and tassel, which were aligned to the SP genome using PASA (v2.0.2) (Haas *et al*., 2003). Finally, the consensus gene models were generated by EvidenceModeler (v1.1.1) and further refined using PASA (v2.0.2) (Haas *et al*., 2008).

### Variant detection

SNPs and small inDels (length < 50 bp) were identified between the popcorn genome and other reference genomes using MUMmer (v4.0) (Marçais *et al*., 2018). Initially, the nucmer tool from MUMmer was employed to generate genome alignments with the following parameters: ‐mum ‐g 1000 ‐c 90 ‐l 40. The alignment files were then filtered using delta‐filter to retain 1‐to‐1 mappings with the parameters ‘‐r ‐q’. SNPs and small inDels were called from these filtered 1‐to‐1 alignment blocks using the show‐snps tool with the ‐Clr parameter. For large structural variations (SVs), ranging from 100 to 100 000 bp, the NUCmer output was further analysed using Assemblytics, a web‐based tool designed for structural variation analysis (Nattestad and Schatz, 2016). Gene synteny blocks were analysed by comparing 1‐to‐1 homologous genes using MCScanX (v1.63) (Wang *et al*., 2012). To visualise the genomic variation, including the number of genes, structural variants (SVs), SNPs, and inDels, the data were plotted using Circos (v0.69‐8) (https://circos.ca/).

### Syntenic block detection

Syntenic regions among the SP, Teosinte, MS71, and B73 (v5) were identified using MCScanX (v1.63). The longest coding sequence (CDS) of each gene from the SP genome was applied for further pairwise comparison. Pairwise high‐confidence sequence alignments were determined for all CDSs from SP, Teosinte, MS71, and B73, using BLASTN with an E‐value threshold of 1e−10. Syntenic blocks were then identified using MCScanX with the syntenic depth of 1‐to‐1, focusing on direct homologous relationships.

### QTL mapping and fine mapping

We genotyped the F_2_ population with 286 lines by 218 single sequence repeat (SSR) markers, evenly distributed on the 10 maize chromosomes. The genetic map was constructed with R/qtl (https://github.com/kbroman/qtl), which spanned 1614 centimorgans (cM) and contained an average genetic distance of 7.4 cM between pairs of neighbouring markers. Phenotypic and genotypic data from 286 F_2_s were imported into R/qtl to detect QTLs using the multiple QTL mapping method. A significance threshold at the *P* = 0.05 level for each trait was determined with 1000 permutations.

To fine‐map the QTL of pericarp colour on chromosome 1, we screened the 240 plants from the F_2:3_ population using markers flanking *P1*. Four recombinant individuals were identified, and the target gene for this QTL was narrowed down within a 3‐Mb region between the two markers 1 k69 and 1 k54 (Figure 2a) on chromosome 1, which harboured the *P1* gene. All primers used for fine mapping are listed in Table S11.

### Comparative mapping

Pairwise genomic comparisons in the locus of the unilateral cross‐incompatibility were conducted using BLASTP (v2.12.0). The syntenic map was generated based on the datasets in Phytozome (https://phytozome‐next.jgi.doe.gov/), including *Zea mays* (B73v5), *Oryza sativa* (v7.0), *Setaria italica* (v2.2), S*orghum bicolor* (v3.1.1) and *Triticum aestivum* (v2.2).

### RNA‐seq analysis

Total RNA was extracted from kernels of SP and MS71 18 days after pollination (18 DAP). DNA‐free RNA samples were used to construct sequencing libraries, which were subsequently sequenced on the Illumina HiSeq 2500 platform, generating a total of 20 Gb of raw sequencing data. The raw RNA‐seq reads were processed using a standard RNA‐seq analysis pipeline (Zhang *et al*., 2019). Initially, the reads were trimmed for quality control and adapter removal using Trimmomatic (v0.39) (https://github.com/usadellab/Trimmomatic). The resulting high‐quality clean reads were then aligned to the SP and MS71 genomes using STAR (v2.7.11b) (Dobin *et al*., 2013). Gene expression levels were quantified as fragments per kilobase of exon per million mapped fragments (FPKM) using Cufflinks and Cuffdiff2 (v2.2) (Trapnell *et al*., 2012).

### Population genetic and phylogenetic analyses of 1467 samples

A comprehensive genotyping‐by‐sequencing dataset of 1467 diverse maize accessions (Table S7) was compiled from publicly available studies. This dataset includes 171 teosinte accessions, 86 popcorn lines, and 1210 tropical and temperate maize lines (Chen *et al*., 2022; Romay *et al*., 2013; Swarts *et al*., 2017). Overlapping SNPs from these samples were merged using BCFtools, and a total of 459 632 SNPs were obtained. This SNP dataset was subsequently utilised for population genetic and phylogenetic analyses.

Principal component analysis (PCA) was performed using GCTA (v1.94.1) with default parameters (Yang *et al*., 2011). Population structure was evaluated using STRUCTURE (v2.3.4) with K‐values ranging from 3 to 20 (Pritchard *et al*., 2000). The optimal number of ancestral populations was finally determined based on the K‐value with the lowest cross‐validation error, calculated with the Evanno method (Evanno *et al*., 2005).

To determine candidate selective sweeps, the cross‐population composite likelihood ratio (XP‐CLR) test (Python version) was applied with a sliding window of 100 kb, a step size of 20 kb, and a maximum of 100 SNPs per window (Chen *et al*., 2010). Genomic regions in the top 5% of XP‐CLR values were recognised as candidate sweeps, and candidate genes within these regions were identified for further analysis.

For phylogenetic analysis, SNPs were initially filtered using VCFtools (maf > 0.1, *r* ^2^ < 0.2, missingness < 0.3), resulting in a reduced dataset of 6725 SNPs (Danecek *et al*., 2011). A maximum‐likelihood phylogenetic tree was constructed for the 1426 germplasm lines using RAxML‐NG with the GTR + G model and an LH epsilon value of 10 (Kozlov *et al*., 2019). The resulting tree was visualised using iTOL (http://itol.embl.de), with genotype classifications and colour‐coding applied as detailed in Table S7.

### Phylogenetic tree of ZmGA1 and ZmPME3

Protein sequences of *ZmGa1* (*Zm00001eb167600*) and *ZmPME3* (*SP_Zm00050172410*) orthologs from *Zea diploperennis* Gigi (*Zd00001aa014509*, *Zd00001aa014504*), *Zea mays parviglumis* Teosinte (*Zv00003aa017981*, *Zv00003aa018483*), *Oryza sativa* (*Os11t0683800*, *Os11t0192400*), *Setaria italica* (*Seita.1G371800*, *Seita.1G371700*), *Sorghum bicolor* (*Sobic.004G350400*, *Sobic.004G350500*) and *Triticum aestivum* (*TraesCS2D02G524000*, *TraesCS6B02G383700*), and the *ZmPMEI4* (*Zm00001eb063520*) as an outgroup were sourced from the MaizeGDB (https://www.maizegdb.org) and Phytozome (http://www.phytozome.net) databases. The protein alignment and phylogenetic tree were analysed using the statistical method of maximum likelihood in MEGA12 (Kumar *et al*., 2024).

### Distribution of the 1.3‐kb insertion in the promoter of *KW1*

The causal site of the 1.3‐kb insertion in the promoter of *KW1* was amplified from 106 teosinte lines and 270 common maize inbred lines using a pair of primers, InDel‐1346 (Table S11). The allele frequencies of the 1.3‐kb insertion were then calculated in teosinte and common maize populations, respectively.

### Measurement of kernel popping‐related phenotypes

Popping volume was evaluated by heating kernels in oil maintained at 200°C, and kernel hardness was measured using a Shore D hardness digital durometer. Each kernel was positioned flat, and hardness measurements were taken from both the front and side surfaces. The highest readings from each surface were recorded, and the average of these values was calculated to represent the kernel's overall hardness. Hardness values from the two NILs (NIL‐N04 and NIL‐D232) were compared using Student's *t*‐test to determine significant differences.

## Author contributions

Z.L. designed the study. X.F., H.L., J.L., Y.S., M.X., X.J., L.D., Q.Z., L.X., G.F., Z.W., Y.Y., and T.F. performed the research. W.L., Y.L., and R.S. contributed new reagents. F.X. and Z.L. analysed the data. F.X. and Z.L. wrote the manuscript.

## Supporting information


**Figure S1** The F_2_ population was derived from crosses between SP and MS71. (a–d) Phenotypic comparisons between the two parents, Strawberry Popcorn (SP) and MS71 from plant architecture (a), ear (b), kernel row number (c), and grain size (d). (e) Genetic map of the SP‐MS71 F_2_ population with 286 individuals. (f) The genotypes of 286 F_2_s. The red, blue and green bars indicate the SP, heterozygous, and MS71 alleles.
**Figure S2** Genome collinearity of chromosome 8 between SP and the parents from the maize NAM population. Genome comparisons reveal the large inversion on chromosome 8. The y‐axis represents SP chromosome 8.
**Figure S3** Venn plot of compared SP genes with B73 and MS71. Genes were compared using blastn with an E‐value threshold of 1e−5, and the best hit was selected.
**Figure S4** The causal site of the key flowering time gene *ZmCCT10* on chromosome 10. Both SP and MS71 contain the 5‐kb insertion in the promoter of *ZmCCT10* at the causal site; thus, the QTL of flowering time corresponding to *ZmCC10* is absent in the F_2_ population between SP and MS71.
**Figure S5** *ZmGa1* gene structure of teosinte. The structure and copy number of genes at the *Ga1* locus in the teosinte (*Zea mays* subsp. *parviglumis*) genome. Grey bars indicate that *Ga1* copies lose gene function, while black bars represent *Ga1* copies that have normal gene function. Blank box, intron; triangle, variants resulting in loss of function.
**Figure S6** Variants in the gene body of *Ga1* among the 22 copies in SP. (a) DNA alignment of *Ga1s* in the gene body in SP. The 1st, 2nd, 4th, 8th, 16th, and 21st *Ga1* copies contain variants (marked in red‐line boxes) inducing a gene‐frame shift or an early stoppage in translation. The 3rd, 5th, 6th, 7th, 11th, 12th, 18th, and 19th copies are truncated. The black‐line box signifies an intron. The start and stop codons are displayed in green boxes. (b) Protein alignment of 14 *GA1* copies with full CDS in SP.
**Figure S7** Comparisons of the *P1* CDSs between SP and MS71. (a) MS71 *Zm00035ab014970* contains a 1‐bp insertion and a 1‐bp deletion (without colour) in the CDS, resulting in a gene‐frame shift. Additionally, other unhighlighted sequences without colour indicate deletions with lengths that are exact multiples of three. (b) Protein alignment of *P1* between SP and MS71.
**Figure S8** Sequence comparisons of the *P1* genes between SP and B73. (a) DNA alignment of the *P1* CDSs between SP and B73. B73 *Zm00001eb014370* harbours a 1‐bp insertion and a 1‐bp deletion (without colour) in the CDS, resulting in a gene‐frame shift. In addition, other uncoloured sequences indicate deletions with lengths that are exact multiples of three. (b) Protein alignment of the *P1* copies between SP and B73.
**Figure S9** *KW1* overexpression lines phenotype. (a, b) *KW1* overexpression lines driven by the ubiquitin promoter. Scale bar, 1 mm. (c) *KW1*‐OE pop upon heating, unlike wild‐type kernels. Scale bar, 1 mm. (d) The *KW1*‐OE exhibits a harder kernel than the wild‐type.
**Figure S10** XP‐CLR top5 genes KEGG enriched terms. Each bubble represents a significantly enriched KEGG pathway, with its size proportional to the number of associated genes. The top five enriched pathways (*P* < 0.05) are displayed based on statistical significance.

**Table S1** Summary of SP genome sequence.
**Table S2** Summary statistics for the popcorn assembly.
**Table S3** BUSCO analysis.
**Table S4** Repetitive elements.
**Table S5** Gene model annotation.
**Table S6** PAVs.
**Table S7** Maize samples for population genomic analysis.
**Table S8** Maize genes under selection based on XP‐CLR tests.
**Table S9** Key genes under selection for the reshaping of popcorn during domestication.
**Table S10** The distribution of ZmKW1 alleles in teosinte and maize populations.
**Table S11** Primers list.

## Data Availability

Data supporting the findings of this work are available within the paper and its supplementary information files. All sequencing data for the creation of the SP have been deposited at the National Genomics Data Center (https://ngdc.cncb.ac.cn/) under the accession number PRJCA034860. The final assembled genome sequence data reported in this paper have been deposited under accession number GWHFILK00000000.1, which is publicly accessible at https://ngdc.cncb.ac.cn/gwh.

## References

[pbi70125-bib-0001] Amor, Y. , Haigler, C.H. , Johnson, S. , Wainscott, M. and Delmer, D.P. (1995) A membrane‐associated form of sucrose synthase and its potential role in synthesis of cellulose and callose in plants. Proc. Natl. Acad. Sci. USA, 92, 9353–9357. 7568131 10.1073/pnas.92.20.9353 PMC40983

[pbi70125-bib-0002] Barazesh, S. and McSteen, P. (2008) Barren inflorescence1 functions in organogenesis during vegetative and inflorescence development in maize. Genetics, 179, 389–401. 18493061 10.1534/genetics.107.084079 PMC2390617

[pbi70125-bib-0003] Becraft, P.W. , Li, K. , Dey, N. and Asuncion‐Crabb, Y. (2002) The maize dek1 gene functions in embryonic pattern formation and cell fate specification. Development, 129, 5217–5225. 12399313 10.1242/dev.129.22.5217

[pbi70125-bib-0004] Bhave, M.R. , Lawrence, S. , Barton, C. and Hannah, L.C. (1990) Identification and molecular characterization of shrunken‐2 cDNA clones of maize. Plant Cell, 2, 581–588. 1967077 10.1105/tpc.2.6.581 PMC159913

[pbi70125-bib-0005] Bolduc, N. and Hake, S. (2009) The maize transcription factor KNOTTED1 directly regulates the gibberellin catabolism gene ga2ox1. Plant Cell, 21, 1647–1658. 19567707 10.1105/tpc.109.068221 PMC2714931

[pbi70125-bib-0006] Bolduc, N. , Yilmaz, A. , Mejia‐Guerra, M.K. , Morohashi, K. , O'Connor, D. , Grotewold, E. and Hake, S. (2012) Unraveling the KNOTTED1 regulatory network in maize meristems. Genes Dev. 26, 1685–1690. 22855831 10.1101/gad.193433.112 PMC3418586

[pbi70125-bib-0007] Booker, J. , Auldridge, M. , Wills, S. , McCarty, D. , Klee, H. and Leyser, O. (2004) MAX3/CCD7 is a carotenoid cleavage dioxygenase required for the synthesis of a novel plant signaling molecule. Curr. Biol. 14, 1232–1238. 15268852 10.1016/j.cub.2004.06.061

[pbi70125-bib-0008] Buckler, E.S. , Holland, J.B. , Bradbury, P.J. , Acharya, C.B. , Brown, P.J. , Browne, C. , Ersoz, E. *et al*. (2009) The genetic architecture of maize flowering time. Science, 325, 714–718. 19661422 10.1126/science.1174276

[pbi70125-bib-0009] Chen, H. , Patterson, N. and Reich, D. (2010) Population differentiation as a test for selective sweeps. Genome Res. 20, 393–402. 20086244 10.1101/gr.100545.109 PMC2840981

[pbi70125-bib-0010] Chen, L. , Luo, J. , Jin, M. , Yang, N. , Liu, X. , Peng, Y. , Li, W. *et al*. (2022) Genome sequencing reveals evidence of adaptive variation in the genus Zea. Nat. Genet. 54, 1736–1745. 36266506 10.1038/s41588-022-01184-y

[pbi70125-bib-0011] Chen, J. , Wang, Z. , Tan, K. , Huang, W. , Shi, J. , Li, T. , Hu, J. *et al*. (2023) A complete telomere‐to‐telomere assembly of the maize genome. Nat. Genet. 55, 1221–1231. 37322109 10.1038/s41588-023-01419-6 PMC10335936

[pbi70125-bib-0012] Cheng, H. , Concepcion, G.T. , Feng, X. , Zhang, H. and Li, H. (2021) Haplotype‐resolved de novo assembly using phased assembly graphs with hifiasm. Nat. Methods, 18, 170–175. 33526886 10.1038/s41592-020-01056-5 PMC7961889

[pbi70125-bib-0013] Chor, B. , Horn, D. , Goldman, N. , Levy, Y. and Massingham, T. (2009) Genomic DNA k‐mer spectra: models and modalities. Genome Biol. 10, 1–10. 10.1186/gb-2009-10-10-r108 PMC2784323 19814784

[pbi70125-bib-0014] Chuck, G. , Whipple, C. , Jackson, D. and Hake, S. (2010) The maize SBP‐box transcription factor encoded by tasselsheath4 regulates bract development and the establishment of meristem boundaries. Development, 137, 1243–1250. 20223762 10.1242/dev.048348

[pbi70125-bib-0015] Danecek, P. , Auton, A. , Abecasis, G. , Albers, C.A. , Banks, E. , DePristo, M.A. , Handsaker, R.E. *et al*. (2011) The variant call format and VCFtools. Bioinformatics, 27, 2156–2158. 21653522 10.1093/bioinformatics/btr330 PMC3137218

[pbi70125-bib-0016] Dobin, A. , Davis, C.A. , Schlesinger, F. , Drenkow, J. , Zaleski, C. , Jha, S. , Batut, P. *et al*. (2013) STAR: ultrafast universal RNA‐seq aligner. Bioinformatics, 29, 15–21. 23104886 10.1093/bioinformatics/bts635 PMC3530905

[pbi70125-bib-0017] Doebley, J.F. , Gaut, B.S. and Smith, B.D. (2006) The molecular genetics of crop domestication. Cell, 127, 1309–1321. 17190597 10.1016/j.cell.2006.12.006

[pbi70125-bib-0018] Dudchenko, O. , Batra, S.S. , Omer, A.D. , Nyquist, S.K. , Hoeger, M. , Durand, N.C. , Shamim, M.S. *et al*. (2017) De novo assembly of the Aedes aegypti genome using Hi‐C yields chromosome‐length scaffolds. Science, 356, 92–95. 28336562 10.1126/science.aal3327 PMC5635820

[pbi70125-bib-0019] Durand, N.C. , Shamim, M.S. , Machol, I. , Rao, S.S. , Huntley, M.H. , Lander, E.S. and Aiden, E.L. (2016) Juicer provides a one‐click system for analyzing loop‐resolution Hi‐C experiments. Cell Syst. 3, 95–98. 27467249 10.1016/j.cels.2016.07.002 PMC5846465

[pbi70125-bib-0020] Evanno, G. , Regnaut, S. and Goudet, J. (2005) Detecting the number of clusters of individuals using the software STRUCTURE: a simulation study. Mol. Ecol. 14, 2611–2620. 15969739 10.1111/j.1365-294X.2005.02553.x

[pbi70125-bib-0021] Eyre‐Walker, A. , Gaut, R.L. , Hilton, H. , Feldman, D.L. and Gaut, B.S. (1998) Investigation of the bottleneck leading to the domestication of maize. Proc. Natl. Acad. Sci. USA, 95, 4441–4446. 9539756 10.1073/pnas.95.8.4441 PMC22508

[pbi70125-bib-0022] Frey, M. , Chomet, P. , Glawischnig, E. , Stettner, C. , Grün, S. , Winklmair, A. , Eisenreich, W. *et al*. (1997) Analysis of a chemical plant defense mechanism in grasses. Science, 277, 696–699. 9235894 10.1126/science.277.5326.696

[pbi70125-bib-0023] Frey, M. , Schullehner, K. , Dick, R. , Fiesselmann, A. and Gierl, A. (2009) Benzoxazinoid biosynthesis, a model for evolution of secondary metabolic pathways in plants. Phytochemistry, 70, 1645–1651. 19577780 10.1016/j.phytochem.2009.05.012

[pbi70125-bib-0024] Fu, L. , Niu, B. , Zhu, Z. , Wu, S. and Li, W. (2012) CD‐HIT: accelerated for clustering the next‐generation sequencing data. Bioinformatics, 28, 3150–3152. 23060610 10.1093/bioinformatics/bts565 PMC3516142

[pbi70125-bib-0025] Grobman, A. , Bonavia, D. , Dillehay, T.D. , Piperno, D.R. , Iriarte, J. and Holst, I. (2012) Preceramic maize from paredones and huaca prieta, Peru. Proc. Natl. Acad. Sci. USA, 109, 1755–1759. 22307642 10.1073/pnas.1120270109 PMC3277113

[pbi70125-bib-0026] Gross, S.M. and Hollick, J.B. (2007) Multiple trans‐sensing interactions affect meiotically heritable epigenetic states at the maize pl1 locus. Genetics, 176, 829–839. 17435245 10.1534/genetics.107.072496 PMC1894611

[pbi70125-bib-0027] Grotewold, E. , Drummond, B.J. , Bowen, B. and Peterson, T. (1994) The myb‐homologous P gene controls phlobaphene pigmentation in maize floral organs by directly activating a flavonoid biosynthetic gene subset. Cell, 76, 543–553. 8313474 10.1016/0092-8674(94)90117-1

[pbi70125-bib-0028] Haas, B.J. , Delcher, A.L. , Mount, S.M. , Wortman, J.R. , Smith, R.K., Jr. , Hannick, L.I. , Maiti, R. *et al*. (2003) Improving the Arabidopsis genome annotation using maximal transcript alignment assemblies. Nucleic Acids Res. 31, 5654–5666. 14500829 10.1093/nar/gkg770 PMC206470

[pbi70125-bib-0029] Haas, B.J. , Salzberg, S.L. , Zhu, W. , Pertea, M. , Allen, J.E. , Orvis, J. , White, O. *et al*. (2008) Automated eukaryotic gene structure annotation using EVidenceModeler and the Program to Assemble Spliced Alignments. Genome Biol. 9, 1–22. 10.1186/gb-2008-9-1-r7 PMC2395244 18190707

[pbi70125-bib-0030] Haberer, G. , Kamal, N. , Bauer, E. , Gundlach, H. , Fischer, I. , Seidel, M.A. , Spannagl, M. *et al*. (2020) European maize genomes highlight intraspecies variation in repeat and gene content. Nat. Genet. 52, 950–957. 32719517 10.1038/s41588-020-0671-9 PMC7467862

[pbi70125-bib-0031] Hable, W. , Oishi, K. and Schumaker, K. (1998) Viviparous‐5 encodes phytoene desaturase, an enzyme essential for abscisic acid (ABA) accumulation and seed development in maize. Mol. Gen. Genet. 257, 167–176. 9491075 10.1007/s004380050636

[pbi70125-bib-0032] Hoseney, R. , Zeleznak, K. and Abdelrahman, A. (1983) Mechanism of popcorn popping. J. Cereal Sci. 1, 43–52.

[pbi70125-bib-0033] Hu, Y. , Colantonio, V. , Müller, B.S. , Leach, K.A. , Nanni, A. , Finegan, C. , Wang, B. *et al*. (2021) Genome assembly and population genomic analysis provide insights into the evolution of modern sweet corn. Nat. Commun. 12, 1227. 33623026 10.1038/s41467-021-21380-4 PMC7902669

[pbi70125-bib-0034] Huang, C. , Sun, H. , Xu, D. , Chen, Q. , Liang, Y. , Wang, X. , Xu, G. *et al*. (2018) ZmCCT9 enhances maize adaptation to higher latitudes. Proc. Natl. Acad. Sci. USA, 115, E334–E341. 29279404 10.1073/pnas.1718058115 PMC5777075

[pbi70125-bib-0035] Huang, Y. , Wang, H. , Zhu, Y. , Huang, X. , Li, S. , Wu, X. , Zhao, Y. *et al*. (2022) THP9 enhances seed protein content and nitrogen‐use efficiency in maize. Nature, 612, 292–300. 36385527 10.1038/s41586-022-05441-2

[pbi70125-bib-0036] Hufford, M.B. , Seetharam, A.S. , Woodhouse, M.R. , Chougule, K.M. , Ou, S. , Liu, J. , Ricci, W.A. *et al*. (2021) De novo assembly, annotation, and comparative analysis of 26 diverse maize genomes. Science, 373, 655–662. 34353948 10.1126/science.abg5289 PMC8733867

[pbi70125-bib-0037] Jiménez, T.J.T. and Nelson, O.E. (1965) A new fourth chromosome gametophyte locus in maize. J. Hered. 56, 259–263.

[pbi70125-bib-0038] Jones, D.F. (1924) Selective fertilization among the gametes from the same individuals. Proc. Natl. Acad. Sci. USA, 10, 218–221. 16586928 10.1073/pnas.10.6.218 PMC1085625

[pbi70125-bib-0039] Jones, Z.G. and Goodman, M.M. (2018) Identification of M‐type gametophyte factors in maize genetic resources. Crop Sci. 58, 719–727.

[pbi70125-bib-0040] Juarez, M.T. , Kui, J.S. , Thomas, J. , Heller, B.A. and Timmermans, M.C. (2004) microRNA‐mediated repression of rolled leaf1 specifies maize leaf polarity. Nature, 428, 84–88. 14999285 10.1038/nature02363

[pbi70125-bib-0041] Kermicle, J.L. (2006) A selfish gene governing pollen‐pistil compatibility confers reproductive isolation between maize relatives. Genetics, 172, 499–506. 16157680 10.1534/genetics.105.048645 PMC1456177

[pbi70125-bib-0042] Kozlov, A.M. , Darriba, D. , Flouri, T. , Morel, B. and Stamatakis, A. (2019) RAxML‐NG: a fast, scalable and user‐friendly tool for maximum likelihood phylogenetic inference. Bioinformatics, 35, 4453–4455. 31070718 10.1093/bioinformatics/btz305 PMC6821337

[pbi70125-bib-0043] Kumar, S. , Stecher, G. , Suleski, M. , Sanderford, M. , Sharma, S. and Tamura, K. (2024) MEGA12: Molecular evolutionary genetic analysis version 12 for adaptive and green computing. Mol. Biol. Evol. 41, msae263. 39708372 10.1093/molbev/msae263 PMC11683415

[pbi70125-bib-0044] La Rocca, N. , Manzotti, P.S. , Cavaiuolo, M. , Barbante, A. , Dalla Vecchia, F. , Gabotti, D. , Gendrot, G. *et al*. (2015) The maize fused leaves1 (fdl1) gene controls organ separation in the embryo and seedling shoot and promotes coleoptile opening. J. Exp. Bot. 66, 5753–5767. 26093144 10.1093/jxb/erv278 PMC4566974

[pbi70125-bib-0045] Li, Y.‐L. , Dong, Y.‐B. , Niu, S.‐Z. and Cui, D.‐Q. (2009) Identification of QTL for popping characteristics using a BC2F2 population and comparison with its F2: 3 population in popcorn. Agric. Sci. China, 8, 137–143.

[pbi70125-bib-0046] Li, P. , Ma, H. , Xiao, N. , Zhang, Y. , Xu, T. and Xia, T. (2023) Overexpression of the ZmSUS1 gene alters the content and composition of endosperm starch in maize (*Zea mays* L.). Planta, 257, 97. 37052727 10.1007/s00425-023-04133-z

[pbi70125-bib-0047] Li, Y. , Wang, J. , Zhong, S. , Huo, Q. , Wang, Q. , Shi, Y. , Liu, H. *et al*. (2024) MADS‐box encoding gene Tunicate1 positively controls maize yield by increasing leaf number above the ear. Nat. Commun. 15, 9799. 39532880 10.1038/s41467-024-54148-7 PMC11557842

[pbi70125-bib-0048] Manicacci, D. , Falque, M. , Le Guillou, S. , Piegu, B. , Henry, A.M. , Le Guilloux, M. , Damerval, C. *et al*. (2007) Maize Sh2 gene is constrained by natural selection but escaped domestication. J. Evol. Biol. 20, 503–516. 17305816 10.1111/j.1420-9101.2006.01264.x

[pbi70125-bib-0049] Marçais, G. , Delcher, A.L. , Phillippy, A.M. , Coston, R. , Salzberg, S.L. and Zimin, A. (2018) MUMmer4: a fast and versatile genome alignment system. PLoS Comput. Biol. 14, e1005944. 29373581 10.1371/journal.pcbi.1005944 PMC5802927

[pbi70125-bib-0050] Miclaus, M. , Wu, Y. , Xu, J.‐H. , Dooner, H.K. and Messing, J. (2011) The maize high‐lysine mutant opaque7 is defective in an acyl‐CoA synthetase‐like protein. Genetics, 189, 1271–1280. 21926304 10.1534/genetics.111.133918 PMC3241421

[pbi70125-bib-0051] Moran Lauter, A.N. , Muszynski, M.G. , Huffman, R.D. and Scott, M.P. (2017) A pectin methylesterase ZmPme3 is expressed in Gametophyte factor1‐s (Ga1‐s) silks and maps to that locus in maize (*Zea mays* L.). Front. Plant Sci. 8, 1926. 29170674 10.3389/fpls.2017.01926 PMC5684833

[pbi70125-bib-0052] Muszynski, M.G. , Moss‐Taylor, L. , Chudalayandi, S. , Cahill, J. , Del Valle‐Echevarria, A.R. , Alvarez‐Castro, I. , Petefish, A. *et al*. (2020) The Maize Hairy Sheath Frayed1 (Hsf1) mutation alters leaf patterning through increased cytokinin signaling. Plant Cell, 32, 1501–1518. 32205456 10.1105/tpc.19.00677 PMC7203929

[pbi70125-bib-0053] Nattestad, M. and Schatz, M.C. (2016) Assemblytics: a web analytics tool for the detection of variants from an assembly. Bioinformatics, 32, 3021–3023. 27318204 10.1093/bioinformatics/btw369 PMC6191160

[pbi70125-bib-0054] Ou, S. , Chen, J. and Jiang, N. (2018) Assessing genome assembly quality using the LTR Assembly Index (LAI). Nucleic Acids Res. 46, e126. 30107434 10.1093/nar/gky730 PMC6265445

[pbi70125-bib-0055] Ou, S. , Su, W. , Liao, Y. , Chougule, K. , Agda, J.R. , Hellinga, A.J. , Lugo, C.S.B. *et al*. (2019) Benchmarking transposable element annotation methods for creation of a streamlined, comprehensive pipeline. Genome Biol. 20, 1–18. 31843001 10.1186/s13059-019-1905-y PMC6913007

[pbi70125-bib-0056] Owens, B.F. , Lipka, A.E. , Magallanes‐Lundback, M. , Tiede, T. , Diepenbrock, C.H. , Kandianis, C.B. , Kim, E. *et al*. (2014) A foundation for provitamin A biofortification of maize: genome‐wide association and genomic prediction models of carotenoid levels. Genetics, 198, 1699–1716. 25258377 10.1534/genetics.114.169979 PMC4256781

[pbi70125-bib-0057] Pritchard, J.K. , Stephens, M. and Donnelly, P. (2000) Inference of population structure using multilocus genotype data. Genetics, 155, 945–959. 10835412 10.1093/genetics/155.2.945 PMC1461096

[pbi70125-bib-0058] Putnam, N.H. , O'Connell, B.L. , Stites, J.C. , Rice, B.J. , Blanchette, M. , Calef, R. , Troll, C.J. *et al*. (2016) Chromosome‐scale shotgun assembly using an in vitro method for long‐range linkage. Genome Res. 26, 342–350. 26848124 10.1101/gr.193474.115 PMC4772016

[pbi70125-bib-0059] Reddy, V.R. , Seshu, G. , Jabeen, F. and Rao, A.S. (2012) Speciality corn types with reference to quality protein Maize (*Zea mays* L.)‐a review. Int. J. Agric. Environ. Biotechnol. 5, 393–400.

[pbi70125-bib-0060] Romay, M.C. , Millard, M.J. , Glaubitz, J.C. , Peiffer, J.A. , Swarts, K.L. , Casstevens, T.M. , Elshire, R.J. *et al*. (2013) Comprehensive genotyping of the USA national maize inbred seed bank. Genome Biol. 14, 1–18. 10.1186/gb-2013-14-6-r55 PMC3707059 23759205

[pbi70125-bib-0061] Simão, F.A. , Waterhouse, R.M. , Ioannidis, P. , Kriventseva, E.V. and Zdobnov, E.M. (2015) BUSCO: assessing genome assembly and annotation completeness with single‐copy orthologs. Bioinformatics, 31, 3210–3212. 26059717 10.1093/bioinformatics/btv351

[pbi70125-bib-0062] Stanke, M. , Keller, O. , Gunduz, I. , Hayes, A. , Waack, S. and Morgenstern, B. (2006) AUGUSTUS: ab initio prediction of alternative transcripts. Nucleic Acids Res. 34, W435–W439. 16845043 10.1093/nar/gkl200 PMC1538822

[pbi70125-bib-0063] Swarts, K. , Gutaker, R.M. , Benz, B. , Blake, M. , Bukowski, R. , Holland, J. , Kruse‐Peeples, M. *et al*. (2017) Genomic estimation of complex traits reveals ancient maize adaptation to temperate North America. Science, 357, 512–515. 28774930 10.1126/science.aam9425

[pbi70125-bib-0064] Sweley, J.C. , Rose, D.J. and Jackson, D.S. (2012) Hybrid and environment effects on popcorn kernel physiochemical properties and their relationship to microwave popping performance. J. Cereal Sci. 55, 188–194.

[pbi70125-bib-0065] Tenaillon, M.I. , U'Ren, J. , Tenaillon, O. and Gaut, B.S. (2004) Selection versus demography: a multilocus investigation of the domestication process in maize. Mol. Biol. Evol. 21, 1214–1225. 15014173 10.1093/molbev/msh102

[pbi70125-bib-0066] Trapnell, C. , Roberts, A. , Goff, L. , Pertea, G. , Kim, D. , Kelley, D.R. , Pimentel, H. *et al*. (2012) Differential gene and transcript expression analysis of RNA‐seq experiments with TopHat and Cufflinks. Nat. Protoc. 7, 562–578. 22383036 10.1038/nprot.2012.016 PMC3334321

[pbi70125-bib-0067] Tzin, V. , Hojo, Y. , Strickler, S.R. , Bartsch, L.J. , Archer, C.M. , Ahern, K.R. , Zhou, S. *et al*. (2017) Rapid defense responses in maize leaves induced by *Spodoptera exigua* caterpillar feeding. J. Exp. Bot. 68, 4709–4723. 28981781 10.1093/jxb/erx274 PMC5853842

[pbi70125-bib-0068] Vázquez‐Carrillo, M.G. , Santiago‐Ramos, D. and de Dios Figueroa‐Cárnas, J. (2019) Kernel properties and popping potential of Chapalote, a Mexican ancient native maize. J. Cereal Sci. 86, 69–76.

[pbi70125-bib-0069] Vollbrecht, E. , Veit, B. , Sinha, N. and Hake, S. (1991) The developmental gene Knotted‐1 is a member of a maize homeobox gene family. Nature, 350, 241–243. 1672445 10.1038/350241a0

[pbi70125-bib-0070] Von Rad, U. , Hüttl, R. , Lottspeich, F. , Gierl, A. and Frey, M. (2001) Two glucosyltransferases are involved in detoxification of benzoxazinoids in maize. Plant J. 28, 633–642. 11851909 10.1046/j.1365-313x.2001.01161.x

[pbi70125-bib-0071] Wang, Y. , Tang, H. , DeBarry, J.D. , Tan, X. , Li, J. , Wang, X. , Lee, T.‐h. *et al*. (2012) MCScanX: a toolkit for detection and evolutionary analysis of gene synteny and collinearity. Nucleic Acids Res. 40, e49. 22217600 10.1093/nar/gkr1293 PMC3326336

[pbi70125-bib-0072] Wang, L. , Beissinger, T.M. , Lorant, A. , Ross‐Ibarra, C. , Ross‐Ibarra, J. and Hufford, M.B. (2017) The interplay of demography and selection during maize domestication and expansion. Genome Biol. 18, 1–13. 29132403 10.1186/s13059-017-1346-4 PMC5683586

[pbi70125-bib-0073] Wang, H. , Huang, Y. , Xiao, Q. , Huang, X. , Li, C. , Gao, X. , Wang, Q. *et al*. (2020) Carotenoids modulate kernel texture in maize by influencing amyloplast envelope integrity. Nat. Commun. 11, 5346. 33093471 10.1038/s41467-020-19196-9 PMC7582188

[pbi70125-bib-0074] Wang, Y. , Li, W. , Wang, L. , Yan, J. , Lu, G. , Yang, N. , Xu, J. *et al*. (2022) Three types of genes underlying the Gametophyte factor1 locus cause unilateral cross incompatibility in maize. Nat. Commun. 13, 4498. 35922428 10.1038/s41467-022-32180-9 PMC9349285

[pbi70125-bib-0075] Wang, B. , Hou, M. , Shi, J. , Ku, L. , Song, W. , Li, C. , Ning, Q. *et al*. (2023) De novo genome assembly and analyses of 12 founder inbred lines provide insights into maize heterosis. Nat. Genet. 55, 312–323. 36646891 10.1038/s41588-022-01283-w

[pbi70125-bib-0076] Wenger, A.M. , Peluso, P. , Rowell, W.J. , Chang, P.‐C. , Hall, R.J. , Concepcion, G.T. , Ebler, J. *et al*. (2019) Accurate circular consensus long‐read sequencing improves variant detection and assembly of a human genome. Nat. Biotechnol. 37, 1155–1162. 31406327 10.1038/s41587-019-0217-9 PMC6776680

[pbi70125-bib-0077] Wu, H. , Galli, M. , Spears, C.J. , Zhan, J. , Liu, P. , Yadegari, R. , Dannenhoffer, J.M. *et al*. (2024) NAKED ENDOSPERM1, NAKED ENDOSPERM2, and OPAQUE2 interact to regulate gene networks in maize endosperm development. Plant Cell, 36, 19–39. 10.1093/plcell/koad247 PMC10734603 37795691

[pbi70125-bib-0078] Xie, Y. , Zhao, Y. , Chen, L. , Wang, Y. , Xue, W. , Kong, D. , Li, C. *et al*. (2024) ZmELF3. 1 integrates the RA2‐TSH4 module to repress maize tassel branching. New Phytol. 241, 490–503. 37858961 10.1111/nph.19329

[pbi70125-bib-0079] Xu, A. , Qiu, J. , Yin, Z. and Wei, C. (2019) Morphological characteristics of endosperm in different regions of maize kernels with different vitreousness. J. Cereal Sci. 87, 273–279.

[pbi70125-bib-0080] Yang, J. , Lee, S.H. , Goddard, M.E. and Visscher, P.M. (2011) GCTA: a tool for genome‐wide complex trait analysis. Am. J. Hum. Genet. 88, 76–82. 21167468 10.1016/j.ajhg.2010.11.011 PMC3014363

[pbi70125-bib-0081] Yang, Q. , Li, Z. , Li, W. , Ku, L. , Wang, C. , Ye, J. , Li, K. *et al*. (2013) CACTA‐like transposable element in ZmCCT attenuated photoperiod sensitivity and accelerated the postdomestication spread of maize. Proc. Natl. Acad. Sci. USA, 110, 16969–16974. 24089449 10.1073/pnas.1310949110 PMC3801022

[pbi70125-bib-0082] Yang, N. , Liu, J. , Gao, Q. , Gui, S. , Chen, L. , Yang, L. , Huang, J. *et al*. (2019) Genome assembly of a tropical maize inbred line provides insights into structural variation and crop improvement. Nat. Genet. 51, 1052–1059. 31152161 10.1038/s41588-019-0427-6

[pbi70125-bib-0083] Zhang, F. and Peterson, T. (2005) Comparisons of maize pericarp color1 alleles reveal paralogous gene recombination and an organ‐specific enhancer region. Plant Cell, 17, 903–914. 15722466 10.1105/tpc.104.029660 PMC1069707

[pbi70125-bib-0084] Zhang, Z. , Zhang, B. , Chen, Z. , Zhang, D. , Zhang, H. , Wang, H. , Zhang, Y.e. *et al*. (2018) A PECTIN METHYLESTERASE gene at the maize Ga1 locus confers male function in unilateral cross‐incompatibility. Nat. Commun. 9, 3678. 30202064 10.1038/s41467-018-06139-8 PMC6131150

[pbi70125-bib-0085] Zhang, X. , Lin, Z. , Wang, J. , Liu, H. , Zhou, L. , Zhong, S. , Li, Y. *et al*. (2019) The tin1 gene retains the function of promoting tillering in maize. Nat. Commun. 10, 5608. 31811145 10.1038/s41467-019-13425-6 PMC6898233

[pbi70125-bib-0086] Zhang, Z. , Li, K. , Zhang, H. , Wang, Q. , Zhao, L. , Liu, J. and Chen, H. (2023) A single silk‐and multiple pollen‐expressed PMEs at the Ga1 locus modulate maize unilateral cross‐incompatibility. J. Integr. Plant Biol. 65, 1344–1355. 36621865 10.1111/jipb.13445

[pbi70125-bib-0087] Zhang, L. , Fu, M. , Li, W. , Dong, Y. , Zhou, Q. , Wang, Q. , Li, X. *et al*. (2024) Genetic variation in ZmKW1 contributes to kernel weight and size in dent corn and popcorn. Plant Biotechnol. J. 22, 1453–1467. 38163293 10.1111/pbi.14279 PMC11123423

