# Supplementary material for: Genome assembly and population genomic analysis reveal the genetic basis of popcorn evolution
Source: Plant Biotechnol J. 2025 May 5;23(7):2911–27. doi: 10.1111/pbi.70125 (PMC12205860; doi:10.1111/pbi.70125)
Supplement: Supplementary file 1 — Figure S1 The F2 population was derived from crosses between SP and MS71. (a–d) Phenotypic comparisons between the two parents, Strawberry Popcorn (SP) and MS71 from plant architecture (a), ear (b), kernel row number (c), and grain size (d). (e) Genetic map of the SP‐MS71 F2 population with 286 individuals. (f) The genotypes of 286 F2s. The red, blue and green bars indicate the SP, heterozygous, and MS71 alleles. Figure S2 Genome collinearity of chromosome 8 between SP and the parents from the maize NAM population. Genome comparisons reveal the large inversion on chromosome 8. The y‐axis represents SP chromosome 8. Figure S3 Venn plot of compared SP genes with B73 and MS71. Genes were compared using blastn with an E‐value threshold of 1e−5, and the best hit was selected. Figure S4 The causal site of the key flowering time gene ZmCCT10 on chromosome 10. Both SP and MS71 contain the 5‐kb insertion in the promoter of ZmCCT10 at the causal site; thus, the QTL of flowering time corresponding to ZmCC10 is absent in the F2 population between SP and MS71. Figure S5 ZmGa1 gene structure of teosinte. The structure and copy number of genes at the Ga1 locus in the teosinte (Zea mays subsp. parviglumis) genome. Grey bars indicate that Ga1 copies lose gene function, while black bars represent Ga1 copies that have normal gene function. Blank box, intron; triangle, variants resulting in loss of function. Figure S6 Variants in the gene body of Ga1 among the 22 copies in SP. (a) DNA alignment of Ga1s in the gene body in SP. The 1st, 2nd, 4th, 8th, 16th, and 21st Ga1 copies contain variants (marked in red‐line boxes) inducing a gene‐frame shift or an early stoppage in translation. The 3rd, 5th, 6th, 7th, 11th, 12th, 18th, and 19th copies are truncated. The black‐line box signifies an intron. The start and stop codons are displayed in green boxes. (b) Protein alignment of 14 GA1 copies with full CDS in SP. Figure S7 Comparisons of the P1 CDSs between SP and MS71. (a) MS71 Zm00035ab0149 [file PBI-23-2911-s002.pptx]

## Slide 1
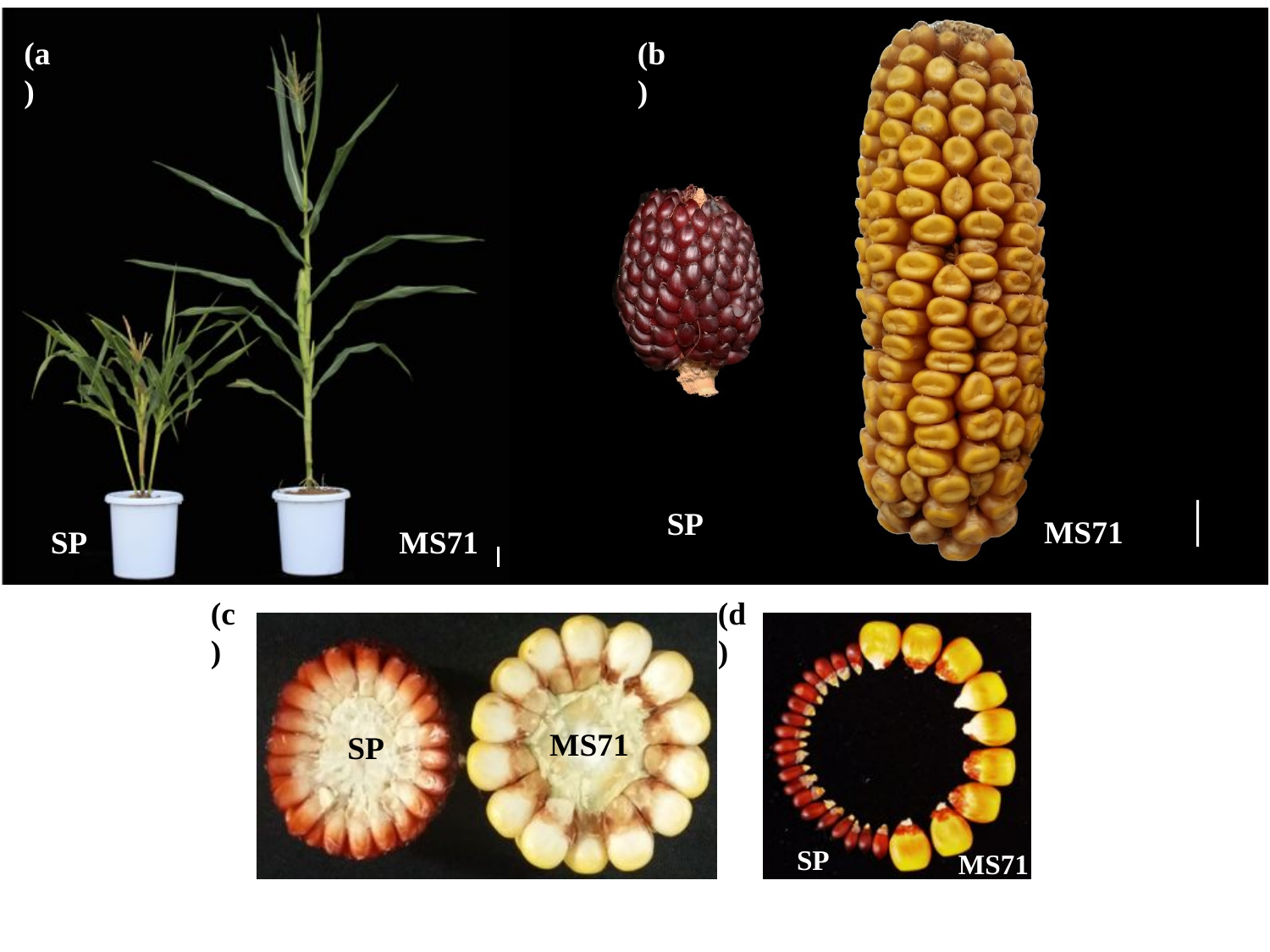

(a)
(b)
SP
MS71
SP
MS71
(c)
(d)
MS71
SP
SP
MS71

## Slide 2
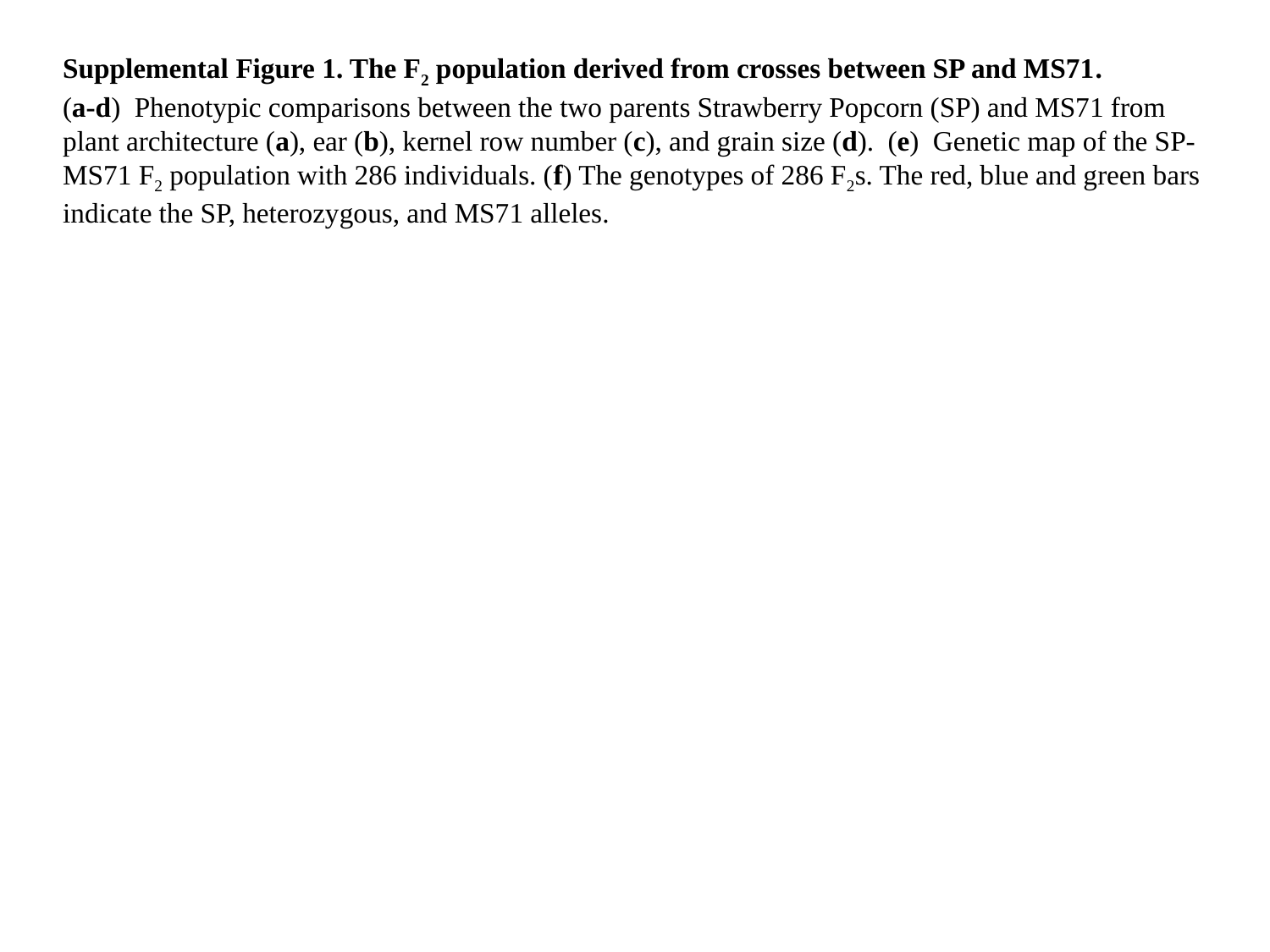

Supplemental Figure 1. The F2 population derived from crosses between SP and MS71.
(a-d) Phenotypic comparisons between the two parents Strawberry Popcorn (SP) and MS71 from plant architecture (a), ear (b), kernel row number (c), and grain size (d). (e) Genetic map of the SP-MS71 F2 population with 286 individuals. (f) The genotypes of 286 F2s. The red, blue and green bars indicate the SP, heterozygous, and MS71 alleles.

## Slide 3
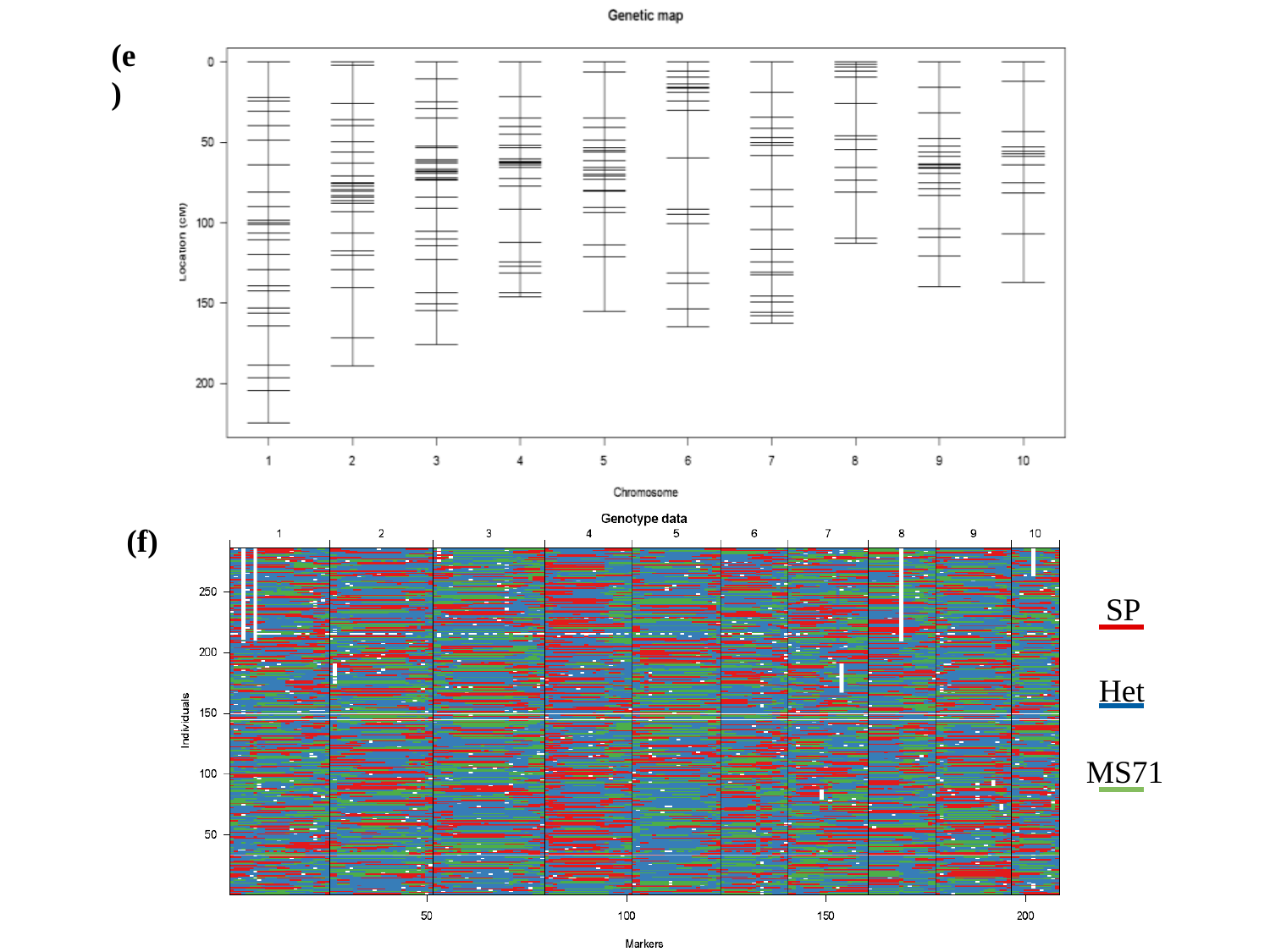

(e)
(f)
SP
Het
MS71

## Slide 4
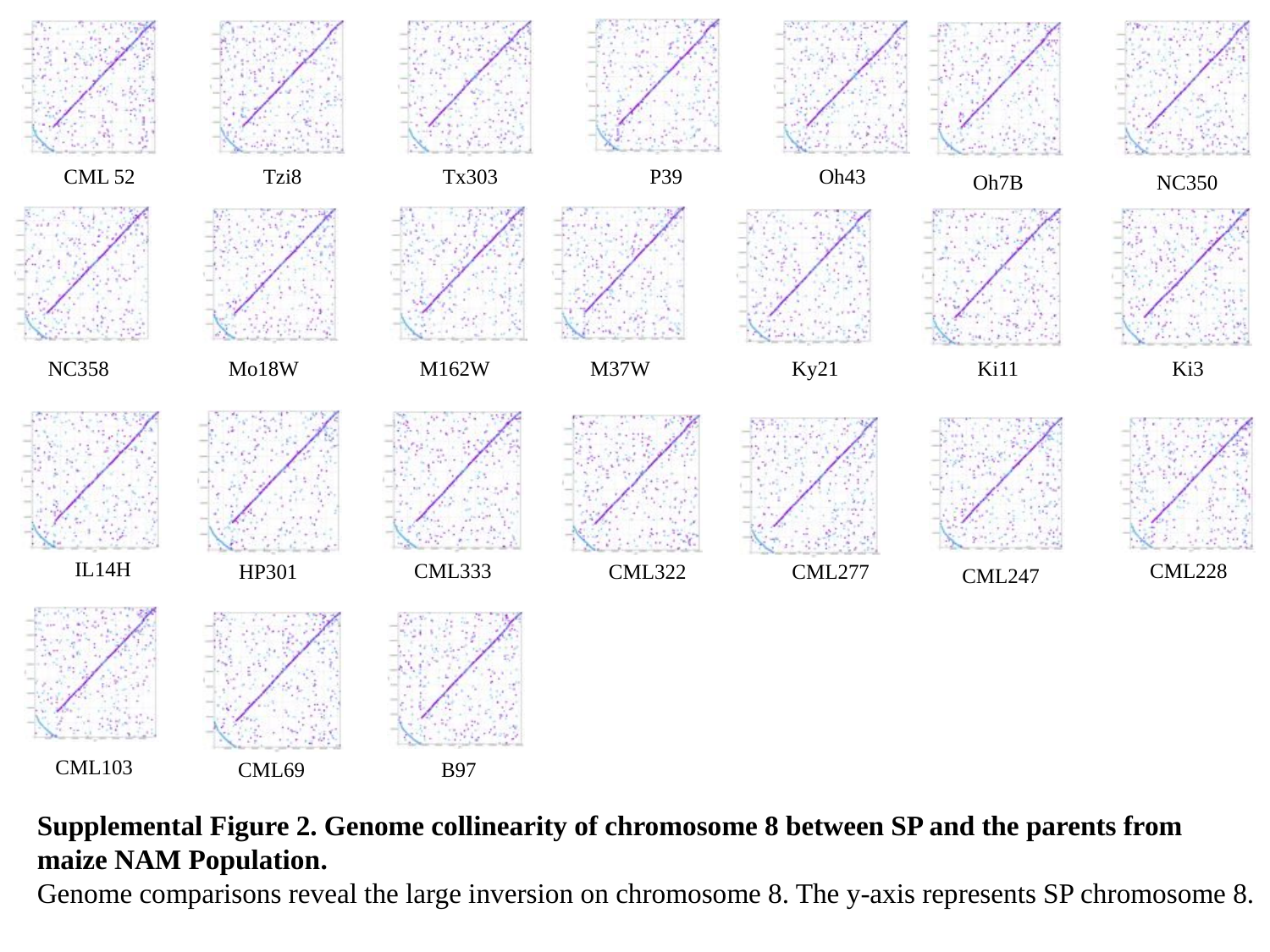

CML 52
Tzi8
Tx303
P39
Oh43
Oh7B
NC350
NC358
Mo18W
M162W
M37W
Ky21
Ki11
Ki3
IL14H
CML228
CML333
HP301
CML322
CML277
CML247
CML103
CML69
B97
Supplemental Figure 2. Genome collinearity of chromosome 8 between SP and the parents from maize NAM Population.
Genome comparisons reveal the large inversion on chromosome 8. The y-axis represents SP chromosome 8.

## Slide 5
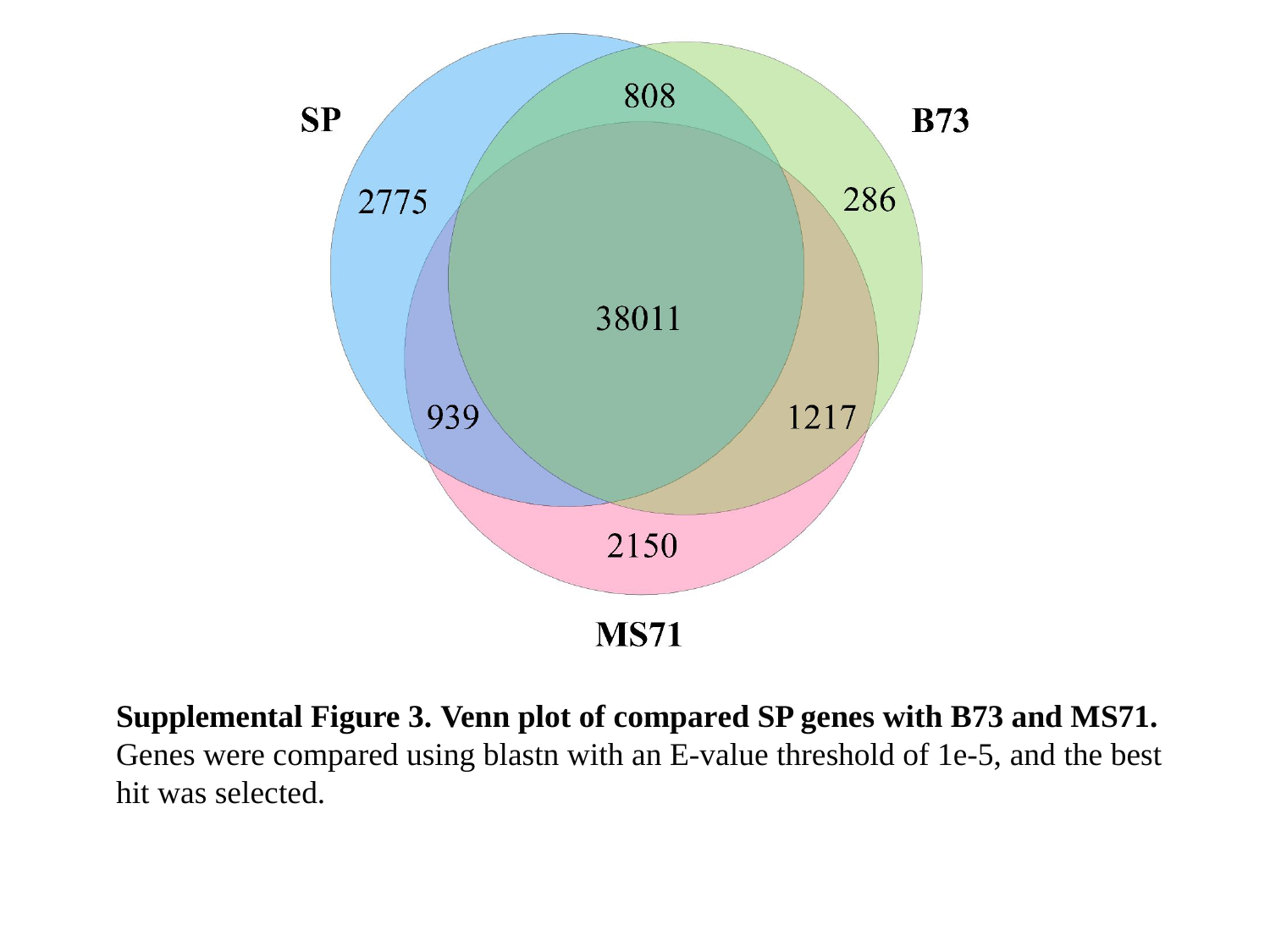

Supplemental Figure 3. Venn plot of compared SP genes with B73 and MS71.
Genes were compared using blastn with an E-value threshold of 1e-5, and the best hit was selected.

## Slide 6
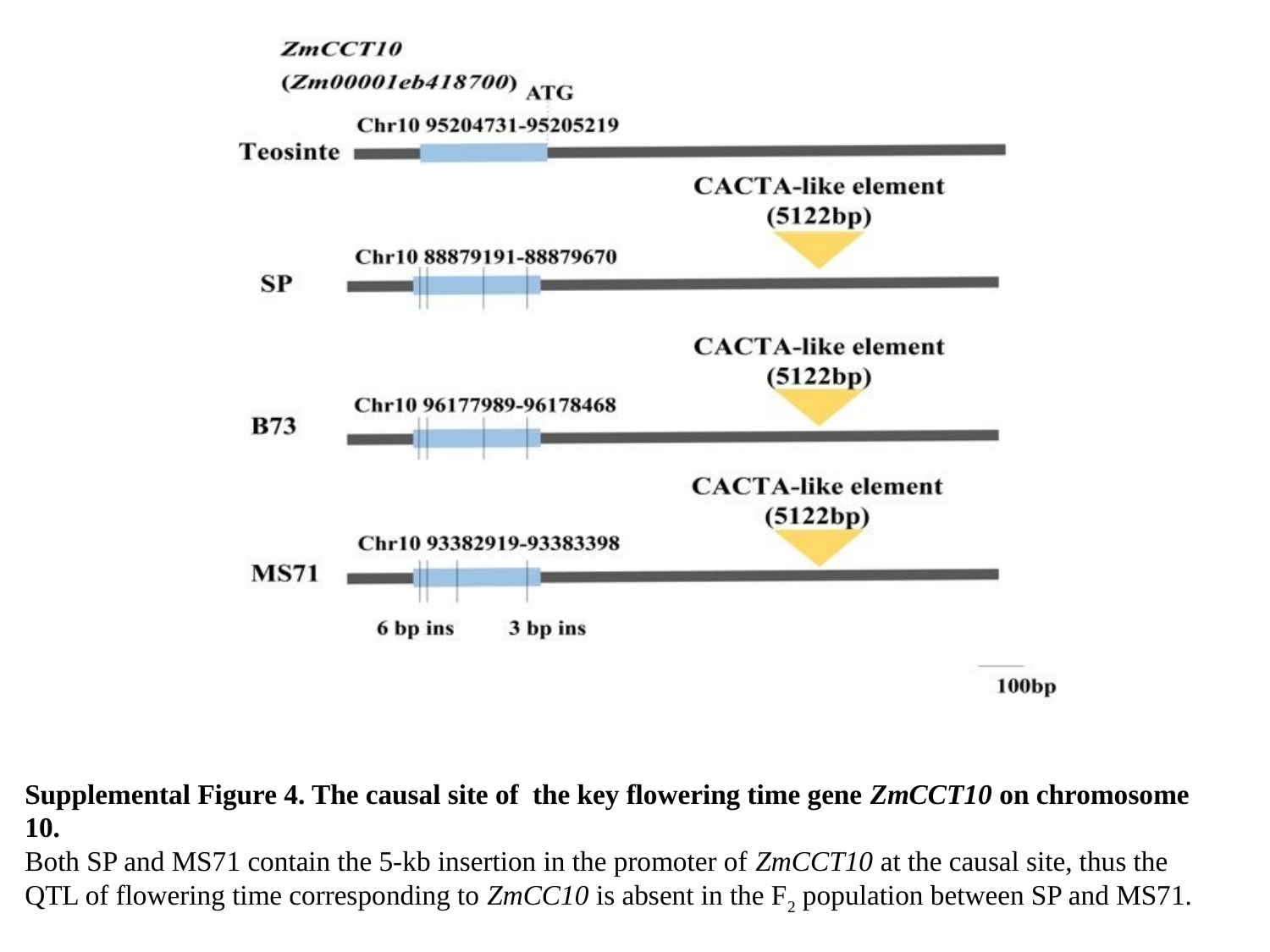

Supplemental Figure 4. The causal site of the key flowering time gene ZmCCT10 on chromosome 10.
Both SP and MS71 contain the 5-kb insertion in the promoter of ZmCCT10 at the causal site, thus the QTL of flowering time corresponding to ZmCC10 is absent in the F2 population between SP and MS71.

## Slide 7
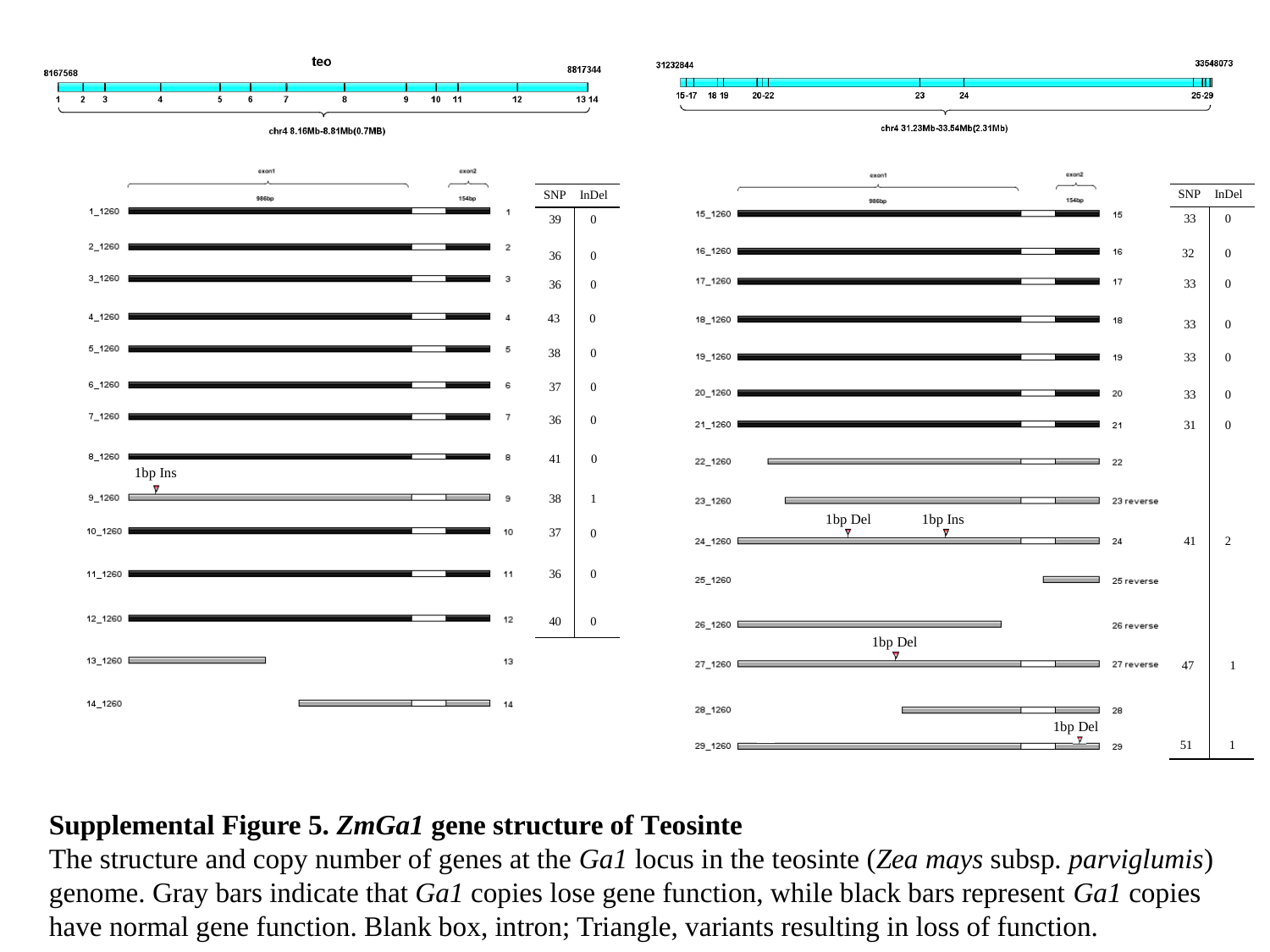

SNP
InDel
SNP
InDel
39
0
36
0
36
0
43
0
38
0
0
37
36
0
41
0
38
1
37
0
36
0
40
0
33
0
0
32
33
0
33
0
33
0
33
0
31
0
1bp Ins
1bp Ins
1bp Del
41
2
1bp Del
47
1
1bp Del
51
1
Supplemental Figure 5. ZmGa1 gene structure of Teosinte
The structure and copy number of genes at the Ga1 locus in the teosinte (Zea mays subsp. parviglumis) genome. Gray bars indicate that Ga1 copies lose gene function, while black bars represent Ga1 copies have normal gene function. Blank box, intron; Triangle, variants resulting in loss of function.

## Slide 8
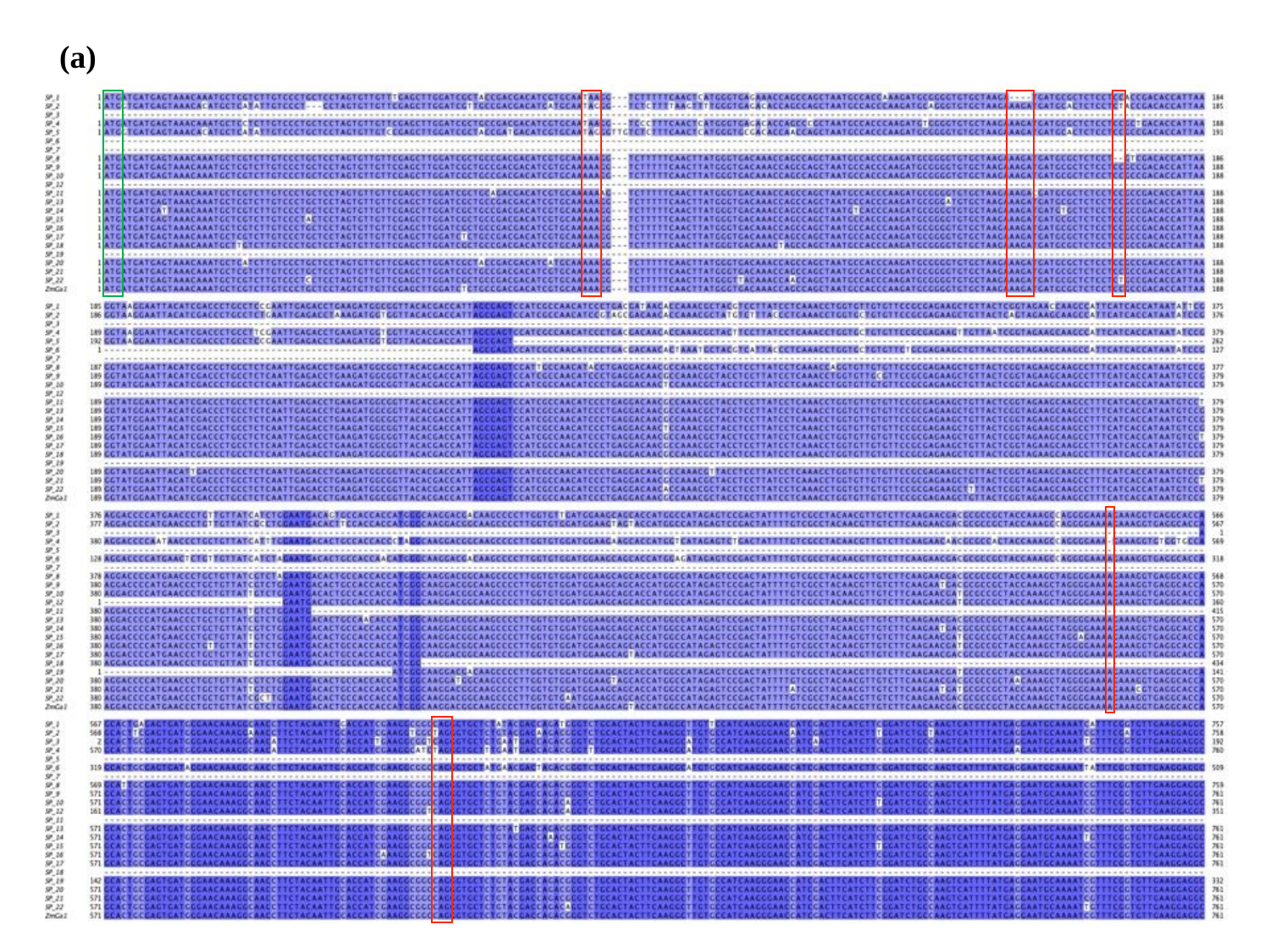

(a)

## Slide 9
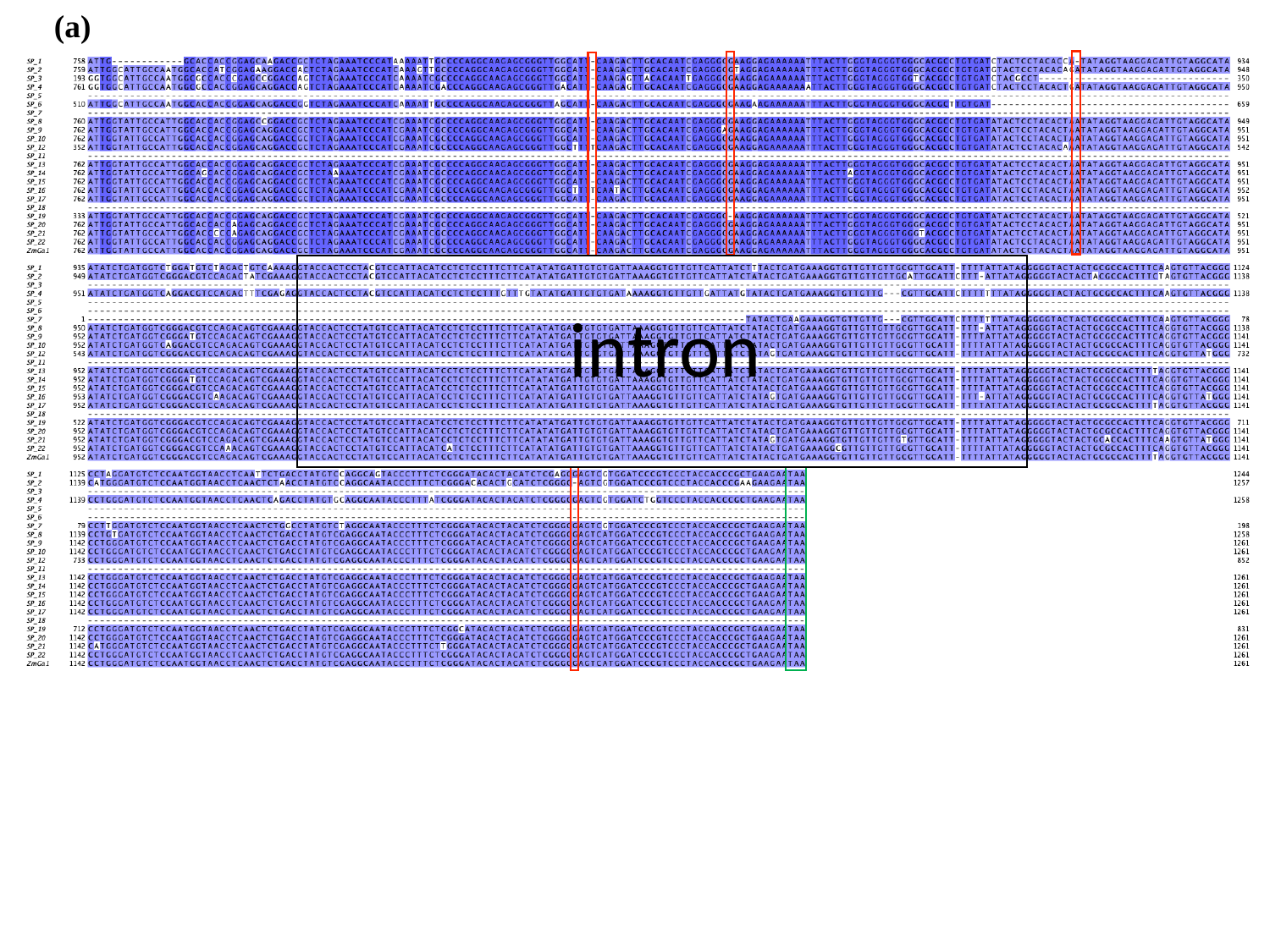

(a)
intron

## Slide 10
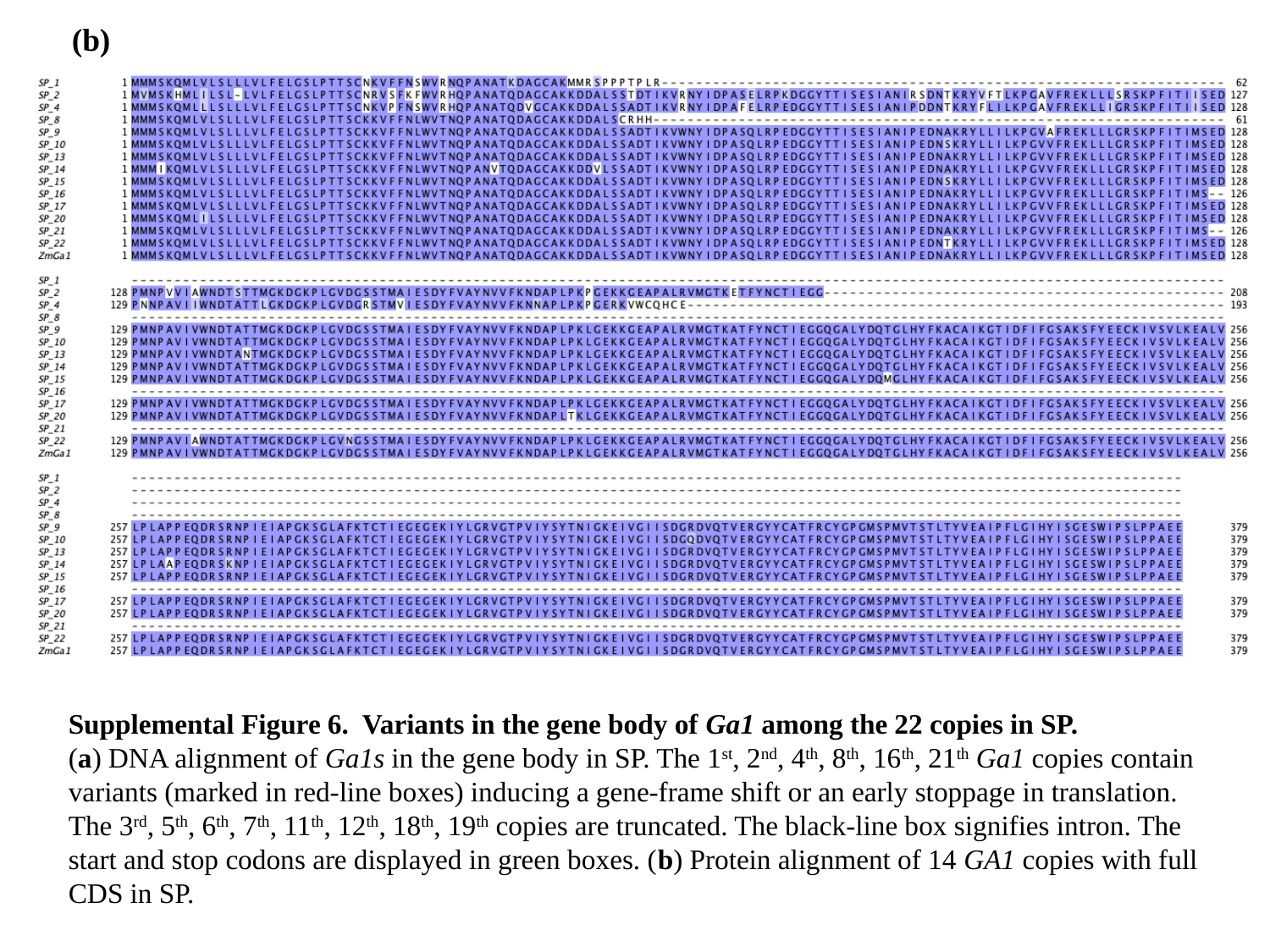

(b)
Supplemental Figure 6. Variants in the gene body of Ga1 among the 22 copies in SP.
(a) DNA alignment of Ga1s in the gene body in SP. The 1st, 2nd, 4th, 8th, 16th, 21th Ga1 copies contain variants (marked in red-line boxes) inducing a gene-frame shift or an early stoppage in translation. The 3rd, 5th, 6th, 7th, 11th, 12th, 18th, 19th copies are truncated. The black-line box signifies intron. The start and stop codons are displayed in green boxes. (b) Protein alignment of 14 GA1 copies with full CDS in SP.

## Slide 11
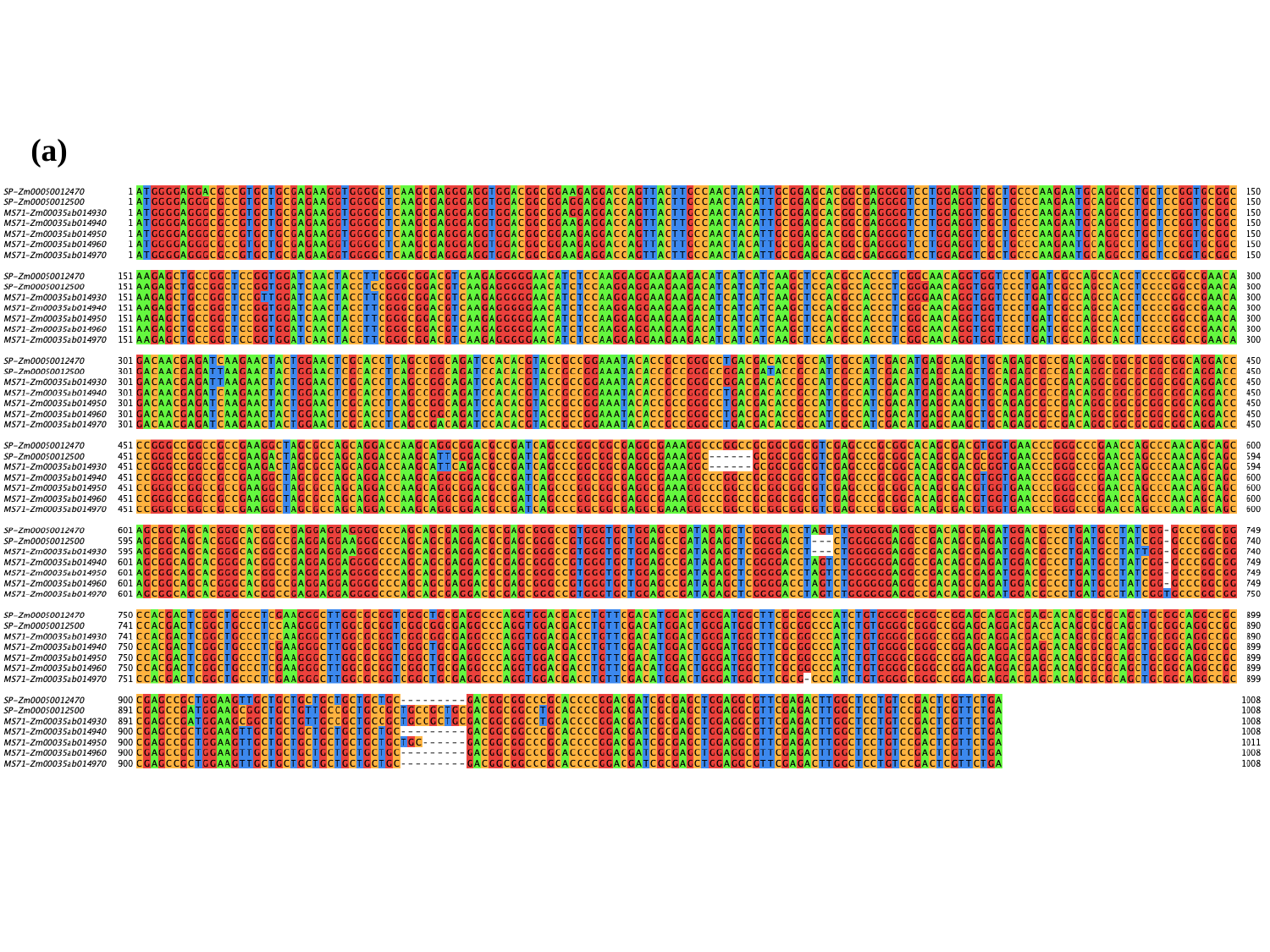

(a)

## Slide 12
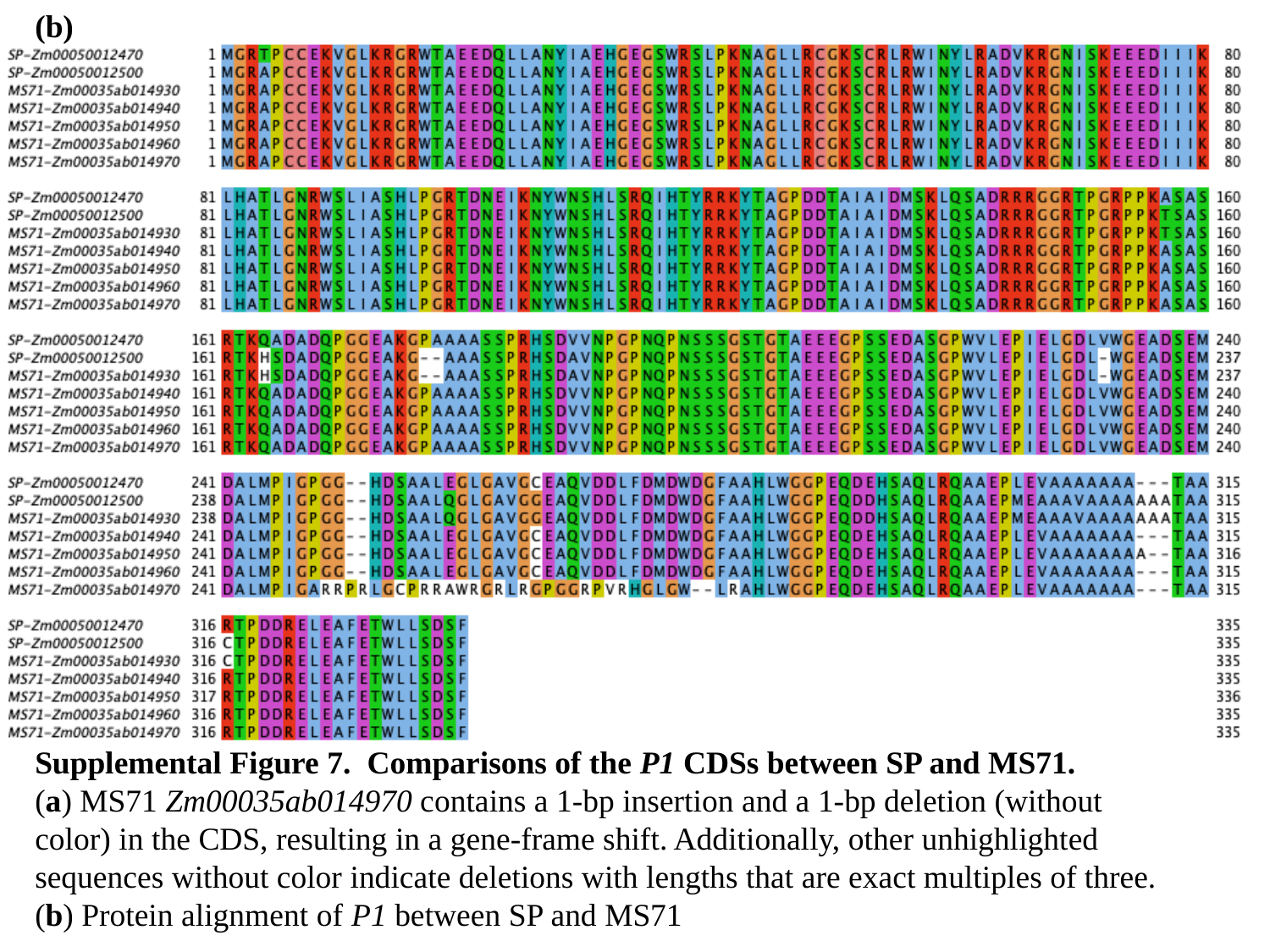

(b)
Supplemental Figure 7. Comparisons of the P1 CDSs between SP and MS71.
(a) MS71 Zm00035ab014970 contains a 1-bp insertion and a 1-bp deletion (without color) in the CDS, resulting in a gene-frame shift. Additionally, other unhighlighted sequences without color indicate deletions with lengths that are exact multiples of three. (b) Protein alignment of P1 between SP and MS71

## Slide 13
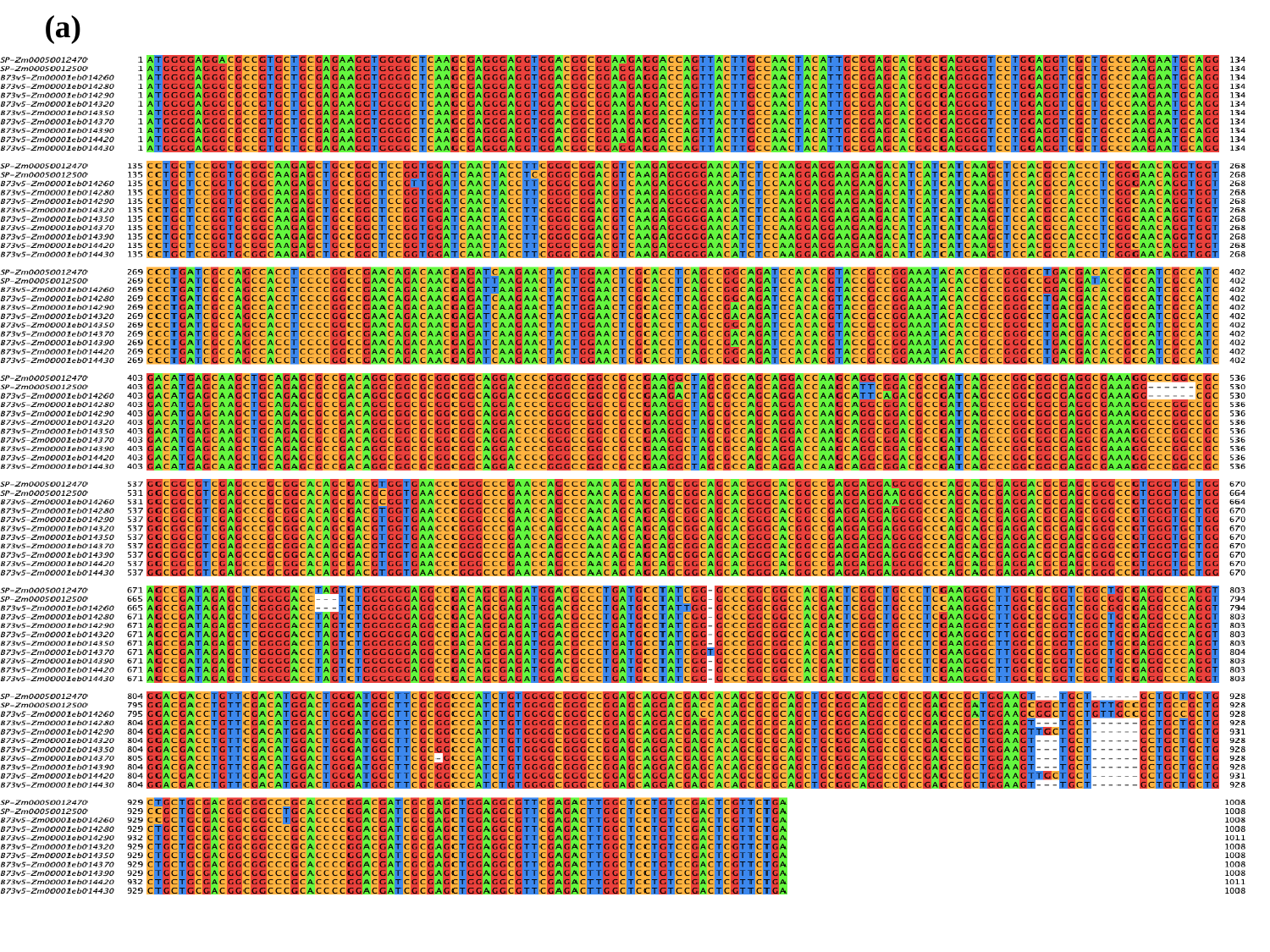

(a)

## Slide 14
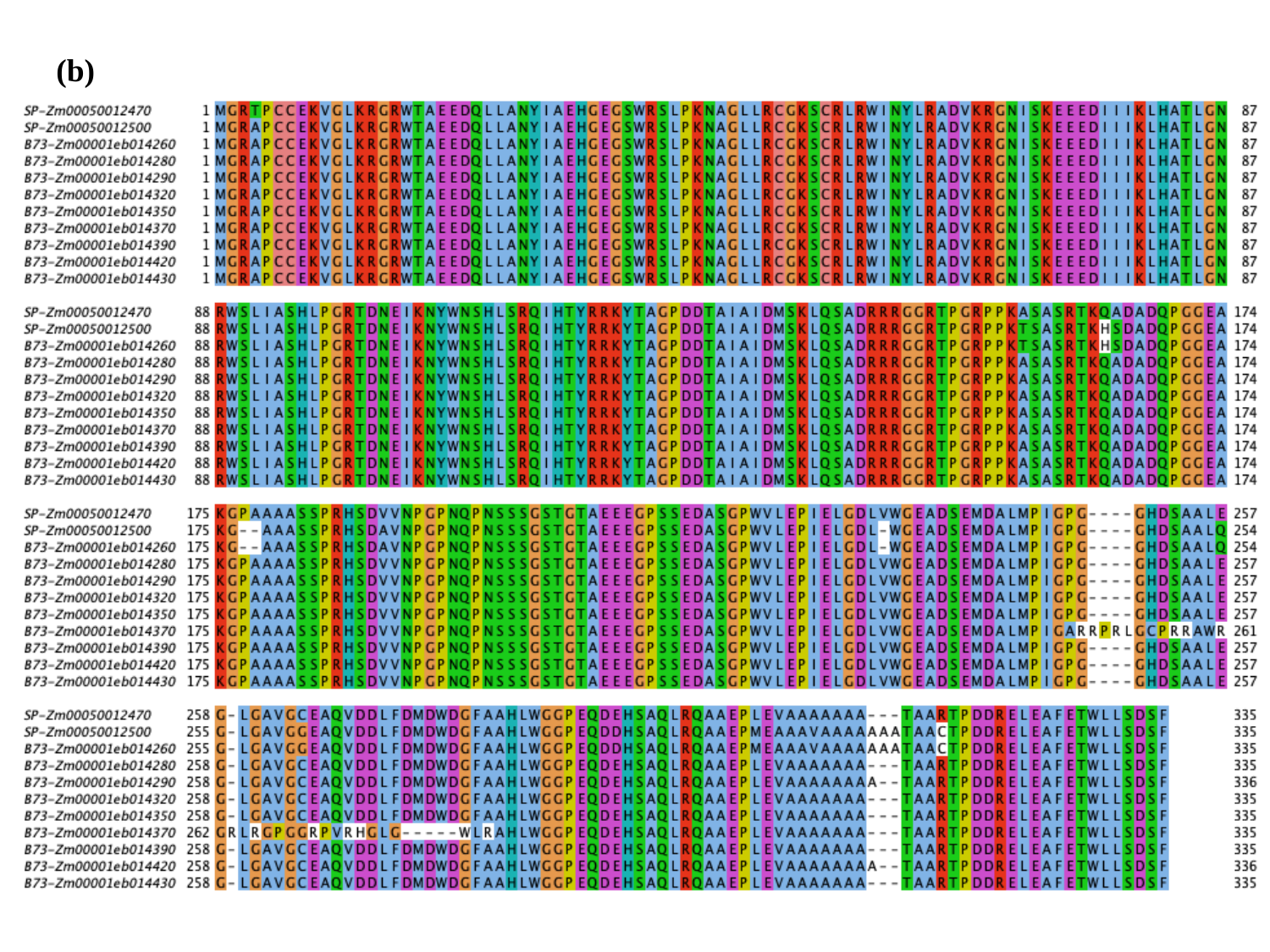

(b)

## Slide 15
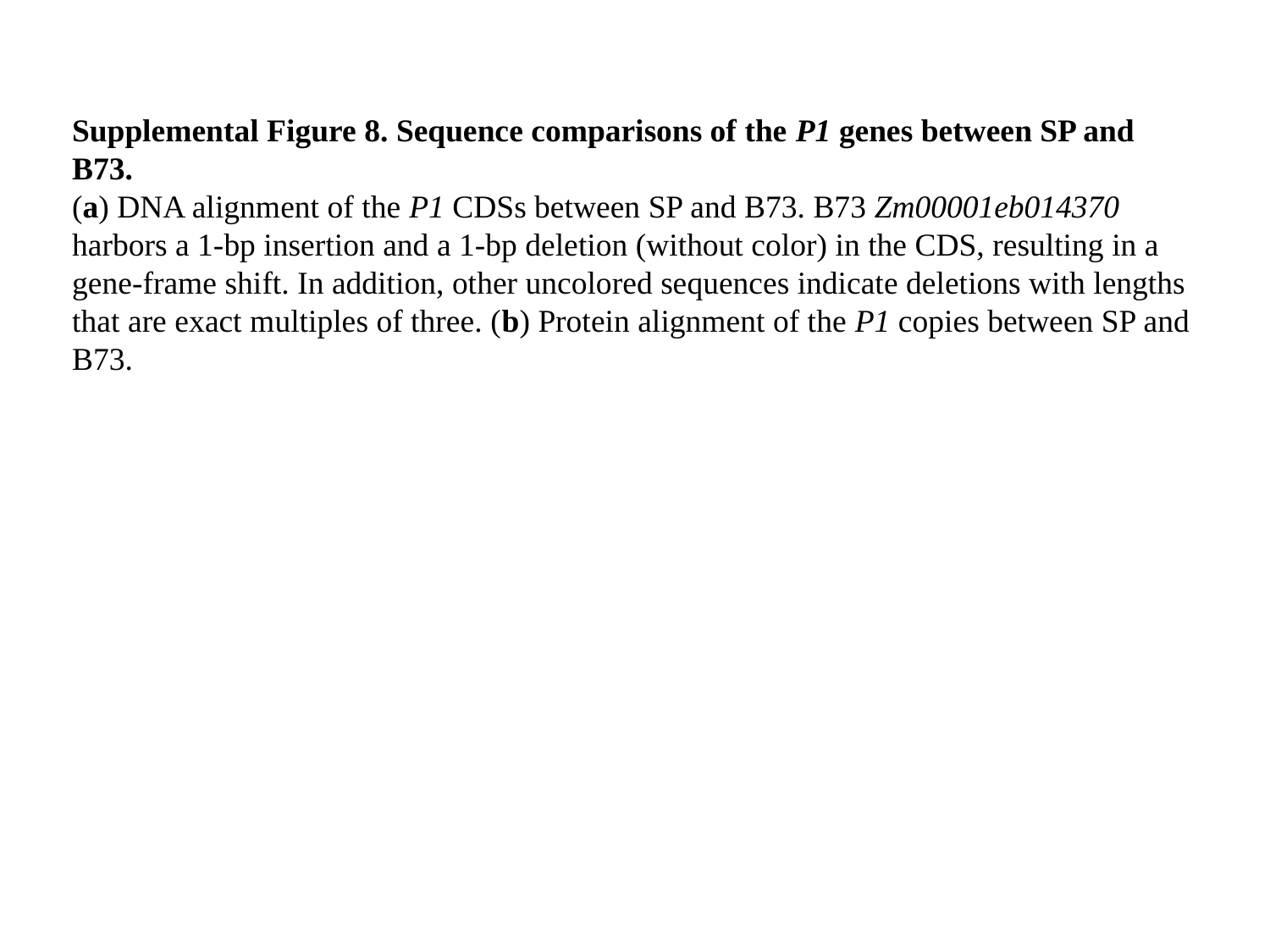

Supplemental Figure 8. Sequence comparisons of the P1 genes between SP and B73.
(a) DNA alignment of the P1 CDSs between SP and B73. B73 Zm00001eb014370 harbors a 1-bp insertion and a 1-bp deletion (without color) in the CDS, resulting in a gene-frame shift. In addition, other uncolored sequences indicate deletions with lengths that are exact multiples of three. (b) Protein alignment of the P1 copies between SP and B73.

## Slide 16
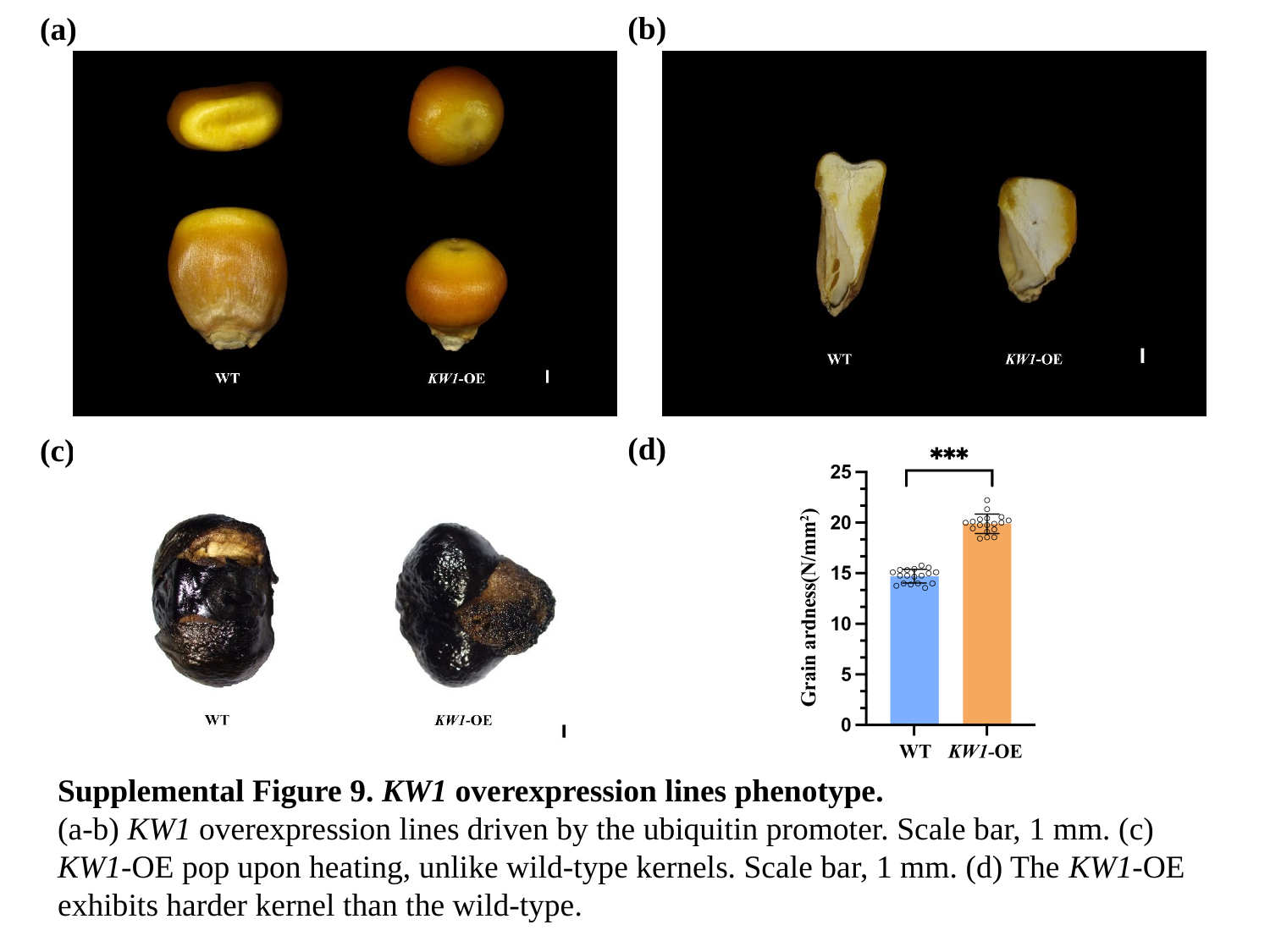

(b)
(a)
(d)
(c)
Supplemental Figure 9. KW1 overexpression lines phenotype.
(a-b) KW1 overexpression lines driven by the ubiquitin promoter. Scale bar, 1 mm. (c) KW1-OE pop upon heating, unlike wild-type kernels. Scale bar, 1 mm. (d) The KW1-OE exhibits harder kernel than the wild-type.

## Slide 17
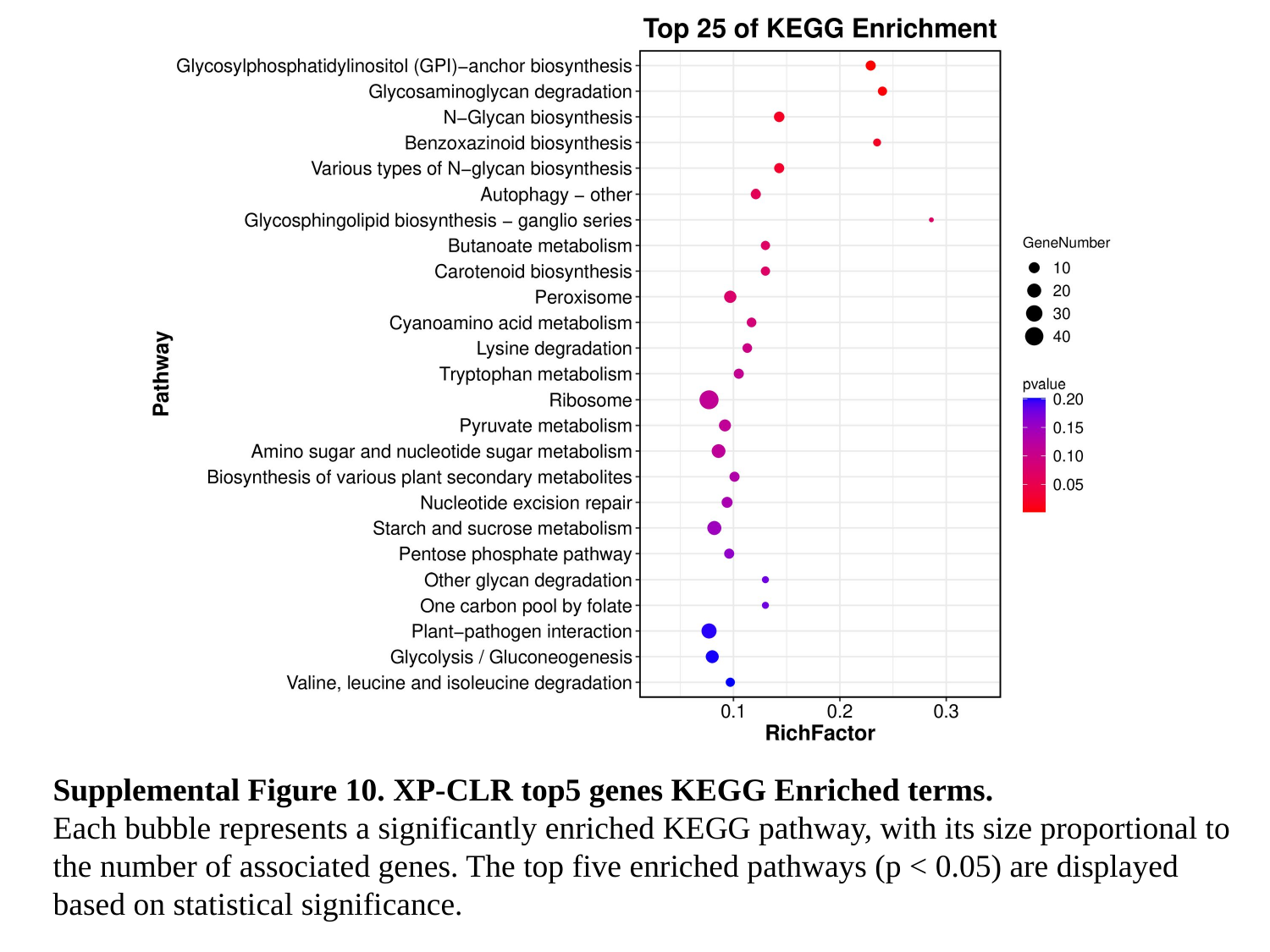

Supplemental Figure 10. XP-CLR top5 genes KEGG Enriched terms.
Each bubble represents a significantly enriched KEGG pathway, with its size proportional to the number of associated genes. The top five enriched pathways (p < 0.05) are displayed based on statistical significance.
